# Targeting a cell state common to triple-negative breast cancers

**DOI:** 10.15252/msb.20145664

**Published:** 2015-02-19

**Authors:** Markus K Muellner, Barbara Mair, Yasir Ibrahim, Claudia Kerzendorfer, Hannelore Lechtermann, Claudia Trefzer, Freya Klepsch, André C Müller, Ernestine Leitner, Sabine Macho-Maschler, Giulio Superti-Furga, Keiryn L Bennett, José Baselga, Uwe Rix, Stefan Kubicek, Jacques Colinge, Violeta Serra, Sebastian MB Nijman

**Affiliations:** 1 CeMM Research Center for Molecular Medicine of the Austrian Academy of SciencesVienna, Austria; 2 Experimental Therapeutics Group, Vall d'Hebron Institute of OncologyBarcelona, Spain; 3 University of Veterinary MedicineVienna, Austria; 4 Memorial Sloan-Kettering Cancer CenterNew York, NY, USA; 5 H. Lee Moffitt Cancer Center and Research InstituteTampa, FL, USA

**Keywords:** breast cancer, cell state, small-molecule screen

## Abstract

Some mutations in cancer cells can be exploited for therapeutic intervention. However, for many cancer subtypes, including triple-negative breast cancer (TNBC), no frequently recurring aberrations could be identified to make such an approach clinically feasible. Characterized by a highly heterogeneous mutational landscape with few common features, many TNBCs cluster together based on their ‘basal-like’ transcriptional profiles. We therefore hypothesized that targeting TNBC cells on a systems level by exploiting the transcriptional cell state might be a viable strategy to find novel therapies for this highly aggressive disease. We performed a large-scale chemical genetic screen and identified a group of compounds related to the drug PKC412 (midostaurin). PKC412 induced apoptosis in a subset of TNBC cells enriched for the basal-like subtype and inhibited tumor growth *in vivo*. We employed a multi-omics approach and computational modeling to address the mechanism of action and identified spleen tyrosine kinase (SYK) as a novel and unexpected target in TNBC. Quantitative phosphoproteomics revealed that SYK inhibition abrogates signaling to STAT3, explaining the selectivity for basal-like breast cancer cells. This non-oncogene addiction suggests that chemical SYK inhibition may be beneficial for a specific subset of TNBC patients and demonstrates that targeting cell states could be a viable strategy to discover novel treatment strategies.

## Introduction

Tumors originating from the mammary gland are the most frequently diagnosed malignancies worldwide and account for 14% of all cancer deaths, making breast cancer the most lethal neoplasm in women (Jemal *et al*, 2011). Breast cancer is a collection of highly heterogeneous neoplasms, and various classification methods have been developed to stratify patient populations. Based on gene expression profiling, at least six major ‘intrinsic’ subtypes have been defined, including luminal A, luminal B, HER2 enriched, basal like, claudin low and normal like (Perou *et al*, 2000; Prat *et al*, 2010). Furthermore, integration of gene expression and copy number variation data has revealed ten clusters that are associated with variable clinical outcomes (Curtis *et al*, 2012). However, even within these breast cancer subtypes, substantial genetic heterogeneity remains, and for many tumors, specific targeted therapies are lacking.

In the clinic, breast tumors are routinely classified based on the presence of the estrogen and progesterone receptors (ER/PR) and HER2/NEU (also known as ERBB2), as expression of these receptors has immediate therapeutic implications. The intrinsic basal-like and claudin-low subtypes are enriched for tumors that do not express these biomarkers (Carey *et al*, 2010; Guiu *et al*, 2012). Indeed, the lack of common ‘druggable’ aberrations in these so-called triple-negative breast cancers makes treatment of these tumors particularly challenging, and despite initial high response rates to classical chemotherapy, women with TNBC have a poor prognosis (Haffty *et al*, 2006; Dent *et al*, 2007) and no FDA-approved targeted treatments are currently available.

Some TNBCs display features of cells that have undergone epithelial to mesenchymal transition (EMT) (Prat *et al*, 2010). EMT is a developmental process in which epithelial cells lose their polarity and acquire a migratory phenotype. In breast cancer, this process has been linked with enhanced tumor-initiating ability, therapeutic resistance, invasiveness and poor prognosis, and basal-like/claudin-low tumors are particularly enriched in EMT traits (Liu *et al*, 2007; Fillmore & Kuperwasser, 2008; Honeth *et al*, 2008; Hennessy *et al*, 2009). The common mRNA expression profile of basal-like breast cancer (BLBC) is indicative of a relatively stable gene expression program (Curtis *et al*, 2012). The existence of this shared and complex molecular ‘cell state’ (Huang, 2011) prompted us to try to exploit this feature pharmacologically using an approach unbiased for target classes.

By employing chemical proteomics, structure–activity relationship studies and computational modeling, next to the previously implicated BLBC target AURKA, we identified the kinase SYK as being critically required for the viability of a subset of breast cancer cells. Inhibition of both AURKA and SYK acted synergistically, suggesting that PKC412 acts by a polypharmacology mechanism of action. Since SYK has never been implicated as a target in BLBCs, we studied this kinase further. SYK is best known as an essential mediator of signaling downstream of a variety of immune receptors, including the B-cell receptor (Turner *et al*, 2000). Constitutive activation of SYK by gene fusion or overexpression has been implicated in hematopoietic malignancies, and SYK inhibitors have shown promise in clinical trials for lymphoma and leukemia (Friedberg *et al*, 2010). In breast cancer, some studies have suggested a tumor suppressive role of SYK (Coopman *et al*, 2000; Sung *et al*, 2009). However, large-scale tumor sequencing studies have not revealed recurrent SYK mutations, and its function in non-hematopoietic cells remains poorly understood.

Surprisingly, in a subset of breast cancer cell lines enriched for the basal-like subtype, SYK inhibition impaired viability by reducing STAT3 activity. SYK inhibition displays hallmarks of non-oncogene addiction (i.e., a vulnerability of cancer cells that is not itself an oncogene; Solimini *et al*, 2007), and our study suggests a clinical application of SYK inhibitors in a molecularly defined subset of breast cancers.

## Results

### A small-molecule screen to identify chemical probes acting on MCF10A-Twist1 cells

The spontaneously immortalized and non-tumorigenic human mammary epithelial cell line MCF10A (ER/PR/HER2 negative, similar to TNBCs) presents pronounced mesenchymal hallmarks upon prolonged ectopic expression of the transcription factor Twist1 (Neve *et al*, 2006; Vesuna *et al*, 2009). Therefore, MCF10A-Twist1 cells provide a model that mimics certain features of post-EMT breast cancer cells, and we subjected this cell line and control MCF10A cells to a small-molecule screen.

We exposed this isogenic cell line pair to a compound library consisting of ∽20,000 chemical structures of considerable diversity, complemented with ∽2,000 FDA-approved drugs, drugs in clinical development, and tool compounds, and measured cell viability 72 h after compound addition. As compounds that killed both cell lines are enriched for general and unspecific toxic agents, we focused on compounds that were specifically toxic to MCF10A-Twist1 cells. Approximately one hundred compounds scored positive in the MCF10A-Twist1 cell-based screen (i.e., were specifically toxic to MCF10A-Twist1 cells compared with MCF10A control cells) and were selected for further analysis (Supplementary Table S1).

**Figure 1:**
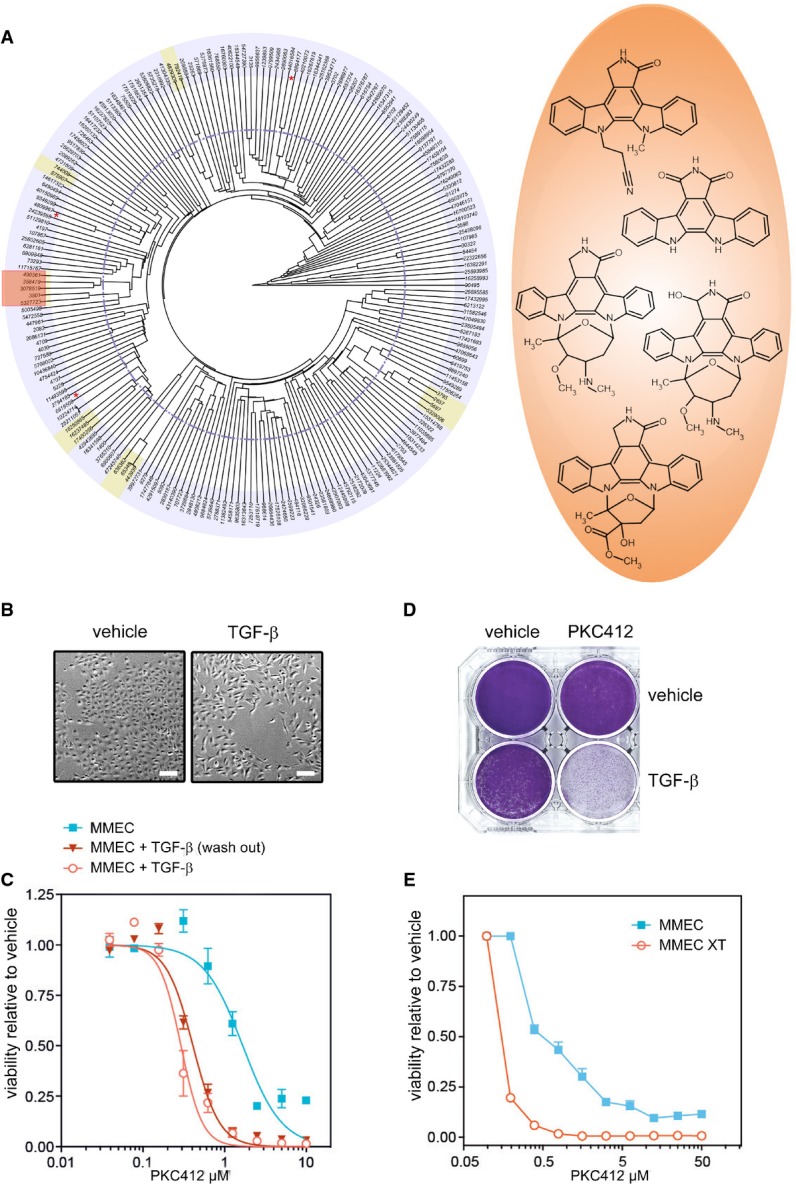
A small-molecule screen identifies PKC412 as a post-EMT breast cancer cell-specific drug A Structural clustering of 104 compounds specifically toxic to MCF10A-Twist1 cells by Tanimoto/Jaccard index. Clusters were selected based on the indicated cutoff (dotted line), and those containing two or more structurally related compounds are highlighted. Compounds clustered with low confidence (based on visual inspection) are indicated by a red asterisk. The chemical structures of the staurosporine-like compound cluster are indicated (orange).

As an initial validation step, we investigated whether specific chemical scaffolds were enriched among the hits using unsupervised clustering of Tanimoto/Jaccard indices for structural similarity, followed by visual inspection. This revealed a group of highly related compounds resembling the natural alkaloid staurosporine (Fig 1A). Staurosporine and many of its derivatives are potent broad-spectrum kinase inhibitors and have been investigated for the treatment of a variety of diseases, including cancer. One such derivative, PKC412 (midostaurin), has advanced into phase III clinical trials for the treatment of acute myeloid leukemia (AML) expressing the mutant tyrosine kinase receptor FLT3/CD135, itself a PKC412 target (Fischer *et al*, 2010). Like staurosporine, PKC412 is a multi-target protein kinase inhibitor and has been shown to have low nanomolar activity against additional kinases including c-KIT, PDGFR and several PKC family members.

B MMEC-HRAS^V12G^ cells treated with TGF-β (10 ng/μl) or vehicle for 14 days to induce a mesenchymal phenotype. Scale bar corresponds to 20 μm.

C PKC412 dose–response curves of cells as in (B) after which TGF-β was washed out for 3 days, and cells with continuous TGF-β treatment for 17 days. Error bars indicate standard deviation. *n* = 3.

D Crystal violet stain of cells from (B) treated with PKC412 (500 nM) for 7 days.

E Dose–response curves of control MMEC-HRAS^V12G^ cells or MMEC-HRAS^V12G^ cells that have undergone EMT *in vivo* (XT) treated with PKC412 for 3 days. Error bars indicate standard deviation. *n* = 3.

### Long-term TGF-β treatment or *in vivo* EMT induces PKC412 sensitivity in murine mammary cancer cells

We investigated whether other post-EMT cell lines were also sensitive for PKC412. We utilized a model consisting of spontaneously immortalized murine mammary epithelial cells (MMECs). These cells become tumorigenic upon mutant HRAS^V12G^ overexpression and acquire a mesenchymal phenotype when treated with the cytokine TGF-β (Fig 1B) (Oft *et al*, 1996). Exposure of these transformed cells to TGF-β for 14 days resulted in a pronounced sensitization to PKC412 by almost an order of magnitude compared to unexposed cells (Fig 1C and D, Supplementary Fig S1). As expected, this effect was not dependent on the continuous presence of TGF-β once cells had undergone EMT, as removal of TGF-β 3 days prior to drug treatment did not significantly alter the response (Fig 1C). We also investigated whether cells that transition to a mesenchymal state *in vivo* would become hypersensitive to PKC412. HRAS^V12G^-expressing MMECs also undergo EMT upon tumor formation in mice, and this mesenchymal phenotype remains stable in cell lines derived from explants (Oft *et al*, 1996). Thus, these explanted cells represent a model for EMT induced in a physiological tumor environment. Consistent with the previous results, the explanted mesenchymal cells were fivefold to tenfold more sensitive to PKC412 than their epithelial counterparts (Fig 1E). Thus, changes in cell state associated with EMT by Twist1 overexpression, chronic stimulation with TGF-β, and *in vivo* EMT result in increased sensitivity to PKC412. This is consistent with the notion that PKC412 targets an epigenetic cell state rather than a somatic mutation.

### Basal-like subtypes are enriched among PKC412-sensitive breast cancer cells *in vitro* and *in vivo*

**Figure 2:**
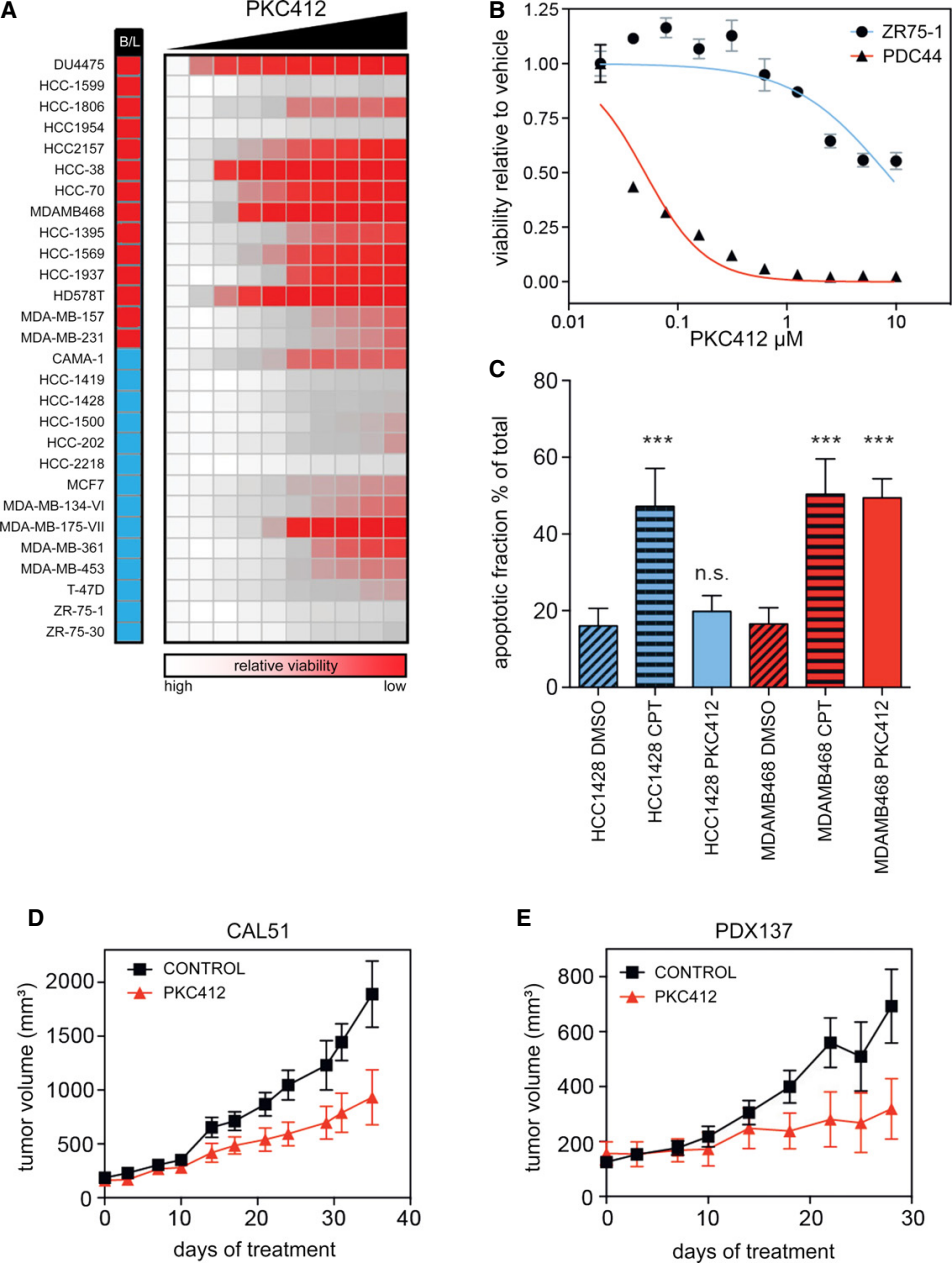
PKC412 targets basal-like breast cancer cells *in vitro* and *in vivo* A Panel of 28 breast cancer cell lines treated with increasing doses (Log2) PKC412 for 5 days. The column after the cell line name indicates intrinsic subtype (blue, luminal; red, basal).

Next, we tested PKC412 cytotoxicity on a panel of 28 breast cancer cell lines representing the major intrinsic subtypes (Neve *et al*, 2006). As basal-like tumors are enriched for mesenchymal features and cell lines have been grouped according to the intrinsic classification, we focused on basal-like and luminal cell lines (Perou *et al*, 2000; Neve *et al*, 2006; Prat *et al*, 2010). Sensitivity varied dramatically across the cell line panel (Fig 2A, Supplementary Dataset S1): Several cell lines were refractory to PKC412 even at the highest concentration tested (50 μM), whereas others were strongly inhibited.

B PKC412 dose–response curve for a low passage patient-derived TNBC cell line (PDC44) and a luminal control cell line (ZR75-1).

C Apoptosis as determined by annexin V staining in basal (red) and luminal (blue) cells treated with PKC412 (500 nM, 16 h) or camptothecin (CPT). ***P < 0.001, ns = not significant. *n* = 3. Statistical significance was analyzed by ANOVA.

D Xenograft model of Cal51 basal breast cancer cells treated with PKC412 for 35 days (oral 100 mg/kg, daily). Shown are the mean and SEM. *P* < 0.01 Akaike information criterion (AIC). *n* ≥ 3.

E Patient-derived xenograft (PDX137) model of a triple-negative breast cancer patient treated with PKC412 (oral 100 mg/kg, daily). Shown are the mean and SEM. *P* < 0.01 (AIC). *n* ≥ 3.

A low passage cell line derived from a TNBC patient (PDC44) with progressive disease despite treatment with various chemotherapeutics, including doxorubicin and cyclophosphamide, was also highly sensitive to PKC412 (Fig 2B).

Dividing the cell lines according to their HER2/ER/PR status revealed that the mean EC_50_ for PKC412 in TNBC cell lines was an order of magnitude lower as compared to receptor-positive cells (Supplementary Fig S4, *P* < 0.001). This is consistent with the notion that EMT-like features contribute to the sensitivity to PKC412, as these are enriched in TNBC. However, three of the nine TNBC cell lines were relatively resistant to PKC412, whereas three of the luminal cell lines displayed an intermediate response, indicating that additional factors may determine the response to the drug.

To investigate the mode of cell death of the sensitive cell lines, we compared the fraction of apoptotic cells in a pair of PKC412-sensitive (i.e., MDA-MB-468) and PKC412-resistant (i.e., HCC1428) cell lines by annexin V staining. Upon exposure to the drug, MDA-MB-468 cells underwent pronounced apoptosis, whereas under identical conditions, HCC1428 cells did not show any response (Fig C and Supplementary Fig S2). This is unlikely due to a general enhanced sensitivity to apoptosis-inducing agents as the topoisomerase inhibitor camptothecin induced apoptosis to a similar extent in both cell lines.

The EC_50_ for PKC412 that we observed for several cell lines was well below the reported steady state plasma concentration in phase I trial patients (0.2–0.7 μM) (Propper *et al*, 2001; Monnerat *et al*, 2004), indicating that this drug is active at a clinically achievable concentration. Therefore, we addressed the potential of PKC412 as a single agent in preclinical *in vivo* tumor models. PKC412 inhibited tumor growth (*P* < 0.01) in the CAL51 xenograft (Fig 2D), a well-defined TNBC/basal-like model refractory to *in vivo* doxorubicin treatment (Coxon *et al*, 2009). We also tested PKC412 in a patient-derived xenograft (PDX) model. PDX models have been described to display original tumor characteristics, such as heterogeneous histology, and thus reproduce additional features of human tumors (DeRose *et al*, 2011; Ibrahim *et al*, 2012). We therefore employed a PDX model derived from a TNBC patient who had displayed progressive disease while treated with FEC90, a combination of 5-FU, epirubicin and cyclophosphamide, in addition to docetaxel neoadjuvant treatment. In accordance with the result obtained with the conventional cell line xenograft, the PDX model displayed a pronounced and significant response to PKC412 (Fig 2E). Thus, PKC412 shows anti-tumor activity *in vivo* and is active against a subset of TNBC tumors. This result is particularly remarkable as staurosporine derivatives such as PKC412 (Borgdorff *et al*, 2014) are multi-kinase inhibitors and their off-target effects therefore may limit their clinical utility. Thus, these results suggest that the identification of the critical target(s) of PKC412 in this setting may provide novel therapeutic opportunities for breast cancer.

### Chemical proteomics identifies candidate kinase targets implicated in PKC412 specificity

**Figure 3:**
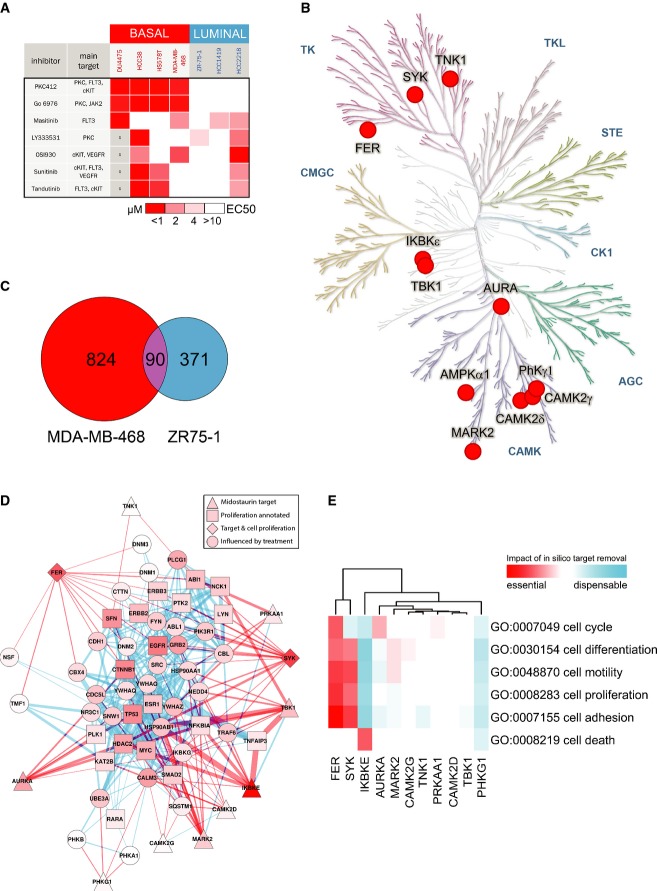
Chemical proteomics and computational modeling highlight SYK as a candidate critical node in basal-like breast cancer cells A A panel of four basal-like and three luminal breast cancer cell lines were treated with the indicated inhibitors targeting canonical PKC412 targets. Red color shading indicates EC_50_. Grayed out boxes indicate cell line/drug combinations that were not tested.

PKC412 has been shown to be a potent inhibitor of several kinases, including FLT3, c-KIT, VEGFR and several PKC family members. Therefore, we investigated whether six structurally unrelated compounds that inhibit various combinations of these ‘canonical’ targets could specifically induce cell death in the basal-like breast cancer cell lines. Only the PKC412 structural analog Go6976 recapitulated the profile, suggesting that a non-canonical target may be responsible for, or at least contribute to, the specificity for TNBC cells (Fig 3A, Supplementary Fig S3 and Supplementary Dataset S2). Of note, the PKC inhibitor LY333531, a compound structurally related to PKC412, did not display the PKC412 cell line specificity pattern, indicating that this small molecule does not inhibit the critical PKC412 target(s) (Fig 3A, Supplementary Fig S4).

B Immobilized PKC412 was used to identify binders in MDA-MB-468 cell lysates by mass spectrometry. Specific and non-specific binders were identified by competition with free PKC412 or the structurally similar but unspecific compound LY333531, respectively. Specific PKC412 binders were mapped onto the kinase tree (solid red circles).

C Venn diagram showing genes differentially expressed (> threefold) upon PKC412 (500 nM) treatment for 6 h in basal-like (MDA-MB-468, red) and luminal (ZR75-1, blue) breast cancer cell lines.

D Most significantly perturbed protein–protein interaction subnetwork (*P* < 0.005) upon PKC412 treatment. Red lines indicate direct interactions with a PKC412 target. Blue lines indicate indirect interactions. Edge thickness indicates simultaneous regulation upon PKC412 treatment (see Materials and Methods).

E Heatmap showing the impact of the individual PKC412 targets on selected GO terms (see also Supplementary Table S3). Red represents reduced association with the GO term, therefore indicating potentially essential targets. Blue represents dispensable targets.

To pursue an unbiased approach for the identification of the relevant PKC412 targets, we performed a chemical proteomic screen using PKC412 for drug affinity purifications (Rix & Superti-Furga, 2009). Immobilized PKC412 was incubated with a cell lysate derived from the sensitive, basal-like MDA-MB-468 cell line, and bound proteins were identified using mass spectrometry. Specificity of the interaction with the PKC412-matrix was assessed by the degree of competition obtained with unbound drug. Targets were considered specific PKC412 interactors if the cognate drug, but not the TNBC non-selective structurally related compound LY333531, efficiently competed matrix retention (Supplementary Table S2). Following these criteria, we identified 11 candidate kinase targets that could contribute to the differential sensitivity profile of PKC412 (Fig 3B).

### Computational modeling highlights SYK as a candidate critical target of PKC412

We next employed a computational approach to generate models of PKC412 mode of action and thereby prioritize hits from the chemical proteomic screen. First, we performed transcriptome analysis by RNA sequencing after a 6-h PKC412 treatment. PKC412-sensitive, basal-like MDA-MB-468 cells displayed a pronounced transcriptional response involving more than 800 genes being up- or down-regulated at least threefold. As expected, and consistent with our previous findings, this gene set was significantly enriched for regulators of apoptosis (Supplementary Fig S5). We observed minimal (∽10%) overlap between the differentially expressed genes with ZR75-1 cells, a PKC412-resistant cell line of the luminal A subtype (Fig 3C). This indicates that these cell lines respond differently to PKC412, resulting in apoptosis predominantly in the basal-like cell line.

Subsequently, we constructed protein–protein interaction networks based on public databases but focusing only on the genes expressed in the cells under investigation (Burkard *et al*, 2010). Both networks were of similar size comprising some 9,000 genes and 104,000 edges representing protein–protein interactions and contained all 11 of our candidate PKC412 targets identified by chemical proteomics, suggesting that the presence or absence of the putative target(s) does not explain selectivity (Supplementary Fig S6). Assuming that concerted expression changes in the network are more likely to represent functional effects (Lage *et al*, 2008), we added edge weights representing gene expression co-regulation upon PKC412 treatment. For all proteins in the network, we used an algorithm mimicking a diffusion process (Kohler *et al*, 2008), starting at the experimentally determined chemical proteomics interactors, to computationally predict the probability of individual nodes being affected by PKC412 treatment over the entire network. The nodes in the MDA-MB-468 network that were most significantly (*P* < 0.005, randomized networks) changed by PKC412 treatment in this quantitative model are indicated in Fig 3D. Among the biological processes most influenced by PKC412 perturbation according to Gene Ontology (GO) terms, we found multiple pathways related to cancer (Colinge *et al*, 2012) (Supplementary Table S3). To determine the impact of each candidate PKC412 target on these enriched GO terms, we recomputed the scores using all but one of the targets (Fig 3E and Supplementary Fig S7). In this analysis, omission of the FER or SYK kinases had a pronounced effect on the enriched GO terms, whereas IKBKE (also known as IKK-epsilon) removal reduced the relationship only with cell death. This suggests that inhibition of these kinases impinges on processes related to proliferation and cell death. Although these three kinases have been previously linked to growth factor signaling and cancer, they have not been linked with a specific breast cancer subtype. Chemical or RNAi-mediated inhibition of IKBKE or FER did not result in specific cytotoxicity (Supplementary Figs S8 and S9), indicating that their inhibition is not sufficient to explain the specificity toward basal-like breast cancer cell lines.

Although Aurora kinase A (AURKA) was only ranked fourth in our network analysis, recent studies suggest that the Aurora/VEGFR inhibitor ENMD-2076 and the pan-Aurora inhibitor AS703569 inhibit the growth of BLBC cells in preclinical models by an unknown mechanism (Romanelli *et al*, 2012; Diamond *et al*, 2013). Indeed, shRNA-mediated and chemical inhibition of AURKA was specifically detrimental to BLBC cell lines (Supplementary Fig S8).

**Figure 4:**
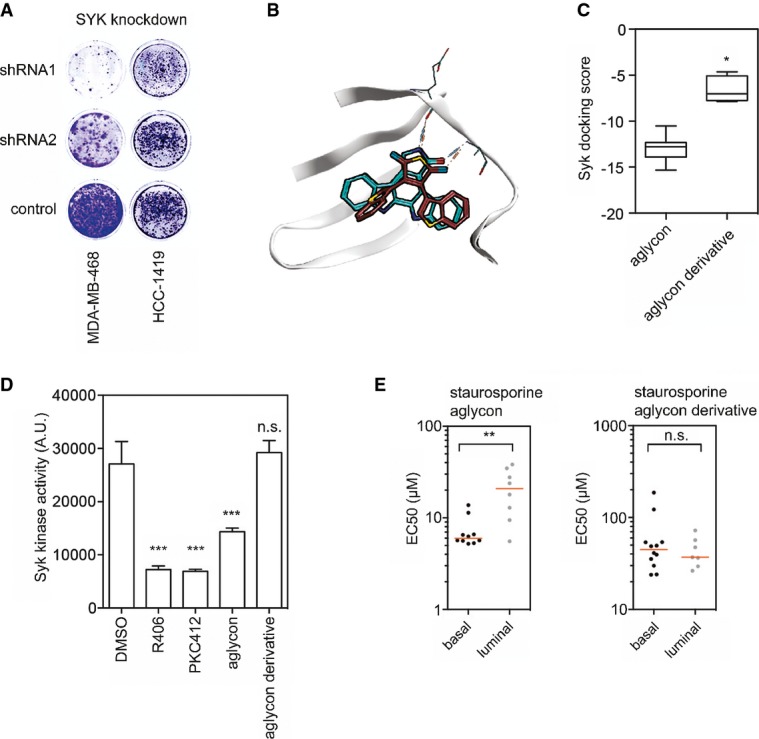
Structure–activity relationship between PKC412 analogs and SYK inhibition A MDA-MD-468 (basal-like) or HCC1419 (luminal) cells were infected with two independent SYK-shRNA or control vectors and plated at equal cell numbers. Plates were stained with crystal violet after 10 days.

In agreement with the chemical proteomics data, PKC412 inhibited SYK autophosphorylation in MDA-MB-468 cells (Supplementary Fig S10). This allows the possibility that SYK inhibition contributes to the mechanism of action of the drug. Indeed, RNAi-mediated depletion of SYK resulted in a pronounced and specific effect (Fig 4A and Supplementary Fig S11). SYK depletion was highly cytotoxic to the basal-like and PKC412-sensitive MDA-MB-468 cells while having a negligible effect on the resistant HCC1419 cell line (Fig 4A). Similar results were obtained in two additional basal-like and luminal cell lines (Supplementary Fig S12). Given that SYK has never been implicated as a drug target in solid tumors and has not been found mutated in breast cancer, we decided to study this interaction further.

B Structural representation of *in silico* docking poses of staurosporine aglycon (cyan) or a derivative lacking the closed central benzene ring (red) into the SYK kinase ATP binding pocket.

C Quantification of docking scores from (B). *P < 0.05, Mann–Whitney *U*-test.

D *In vitro* kinase assay with recombinant SYK kinase incubated with vehicle (DMSO), staurosporine aglycon, staurosporine aglycon derivative or PKC412. The SYK inhibitor R406 was used as a positive control. *n* = 4; error bars indicate standard deviation. ***P < 0.001, Mann–Whitney *U*-test. n.s. = not significant.

E EC_50_ values of a cell line panel treated with the compounds from (C). **P < 0.01. n.s. = not significant.

### SYK is a PKC412 target, and SYK knockdown and chemical inhibition target basal-like breast cancer cells

We performed structure activity relationship (SAR) analysis to further support SYK as a critical PKC412 target in breast cancer cells. Molecular docking of the central scaffold of PKC412 and a derivative into the SYK catalytic pocket indicated that the rigidity provided by the central benzene ring is an important feature for ligand binding (Fig 4B and C). This suggests that PKC412-like compounds that do not have the central benzene ring would not be potent SYK inhibitors and hence not display selectivity for the basal-like breast cancer cell lines. To test this, we synthesized a staurosporine aglycon derivative lacking the benzene ring—3,4-Bis(3-indolyl)-1*H*-pyrrole-2,5-dion, hereafter referred to as ‘staurosporine aglycon derivative’ (Supplementary Fig S13). As predicted, this compound failed to inhibit SYK kinase activity, whereas staurosporine aglycon, PKC412 and the SYK inhibitor R406 (positive control) potently inhibited SYK kinase activity (Fig 4D). Furthermore, only staurosporine aglycon displayed specificity for basal-like cells, whereas the derivative did not (Fig 4E).

**Figure 5:**
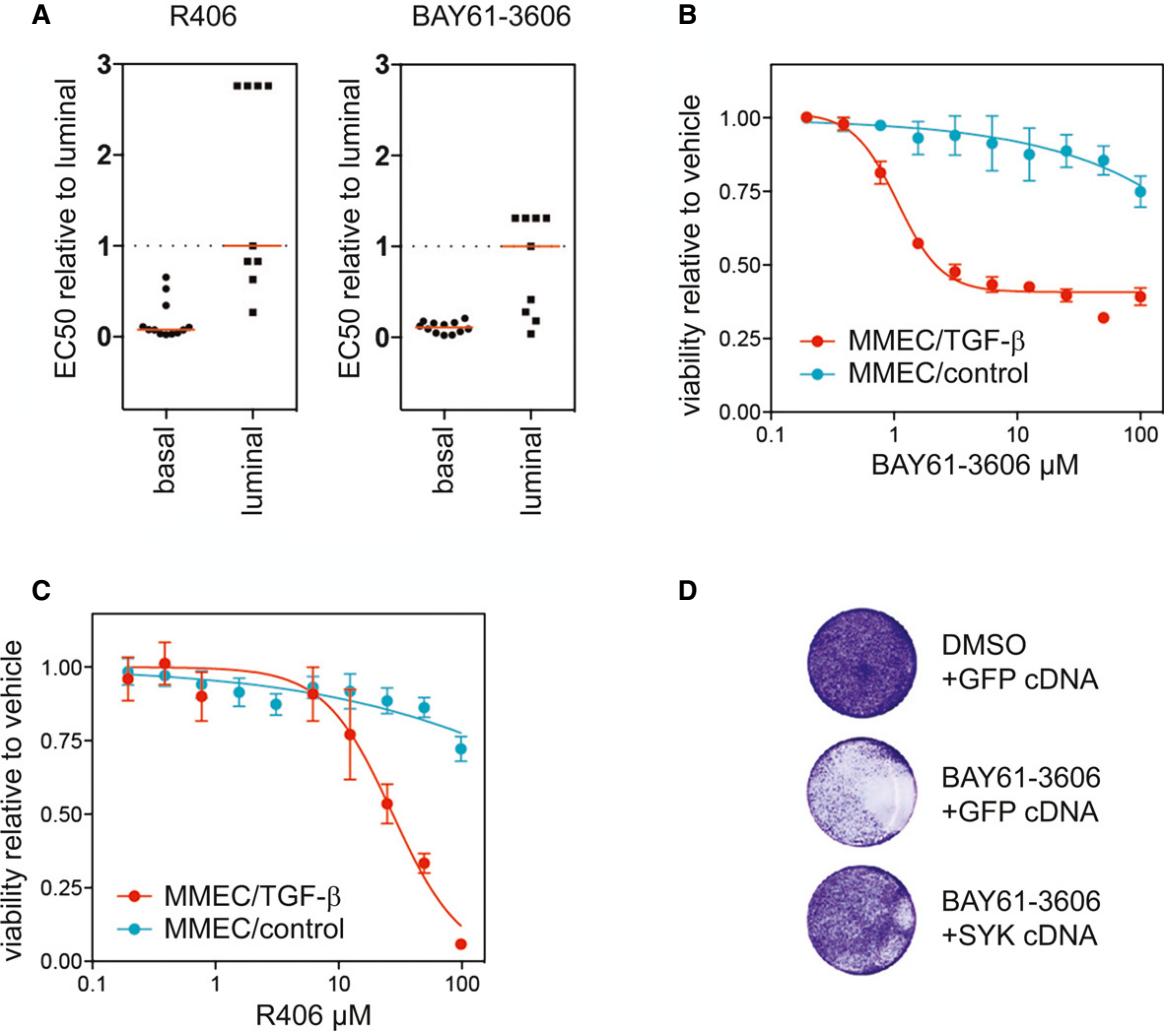
SYK kinase activity is specifically required in basal-like breast cancer cell lines A Cell line panel of basal-like (*n* = 9) and luminal cells (*n* = 10) treated with two SYK inhibitors. *P* < 0.0001 for R406 and *P* < 0.01 for BAY61-6306, Mann–Whitney *U*-test.

R406 and BAY61-3606, two structurally unrelated and selective small-molecule inhibitors of SYK, were also specifically cytotoxic for basal-like cell lines (Fig 5A). These compounds were also more potent in killing the MMEC cells that had undergone EMT induced by TGF-β (Fig 5B and C). Ectopic overexpression of SYK in MDA-MB-468 cells and HCC70 cells partially rescued SYK inhibitor (BAY61-3606) cytotoxicity, which is consistent with the notion that this compound induces toxicity through the inhibition of SYK kinase activity (Fig 5D and Supplementary Figs S14 and S15). Thus, the inhibition of SYK explains at least part of the basal-like TBNC cell line specificity profile of PKC412. Importantly, these experiments reveal the non-oncogene addiction of a subset of breast cancer cell lines to SYK and thereby identify SYK as a novel and critical breast cancer target.

B Dose–response experiment with the SYK inhibitor BAY61-3606 on TGF-β- or vehicle-treated MMEC-HRAS^V12G^ cells. Data are expressed as mean ± SD, *n* = 3.

C Cells as in (B) treated with the SYK inhibitor R406. Data are expressed as mean ± SD, *n* = 3.

D MDA-MB-468 transfected with a SYK cDNA or GFP-expressing vector were treated with 10 μM BAY61-3606 for 7 days. Cells were visualized using crystal violet.

### Quantitative phosphoproteomics reveals STAT3 as a critical SYK effector

**Figure 6:**
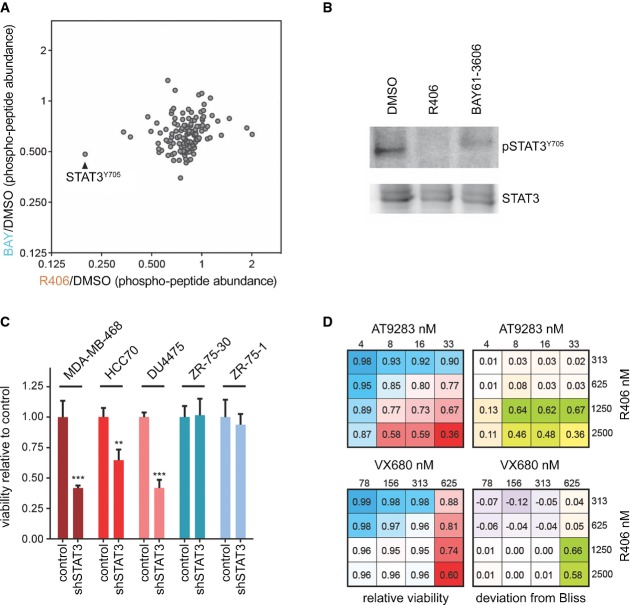
STAT3 is a critical downstream SYK effector A Quantitative phosphoproteomics of MDA-MB-468 cells treated with indicated SYK inhibitors or vehicle for 6 h. Fold change in relative phosphopeptide abundance compared to DMSO is indicated.

To gain insight into the molecular mechanism underlying the critical requirement for SYK activity in basal-like TNBCs, we determined the changes in protein tyrosine phosphorylation by quantitative phosphoproteomics upon chemical inhibition of SYK. MDA-MB-468 cells were exposed to vehicle (DMSO), R406 or BAY61-3606 for 6 h in duplicates. Following cell lysis and protein digestion, tyrosine-phosphorylated proteins were enriched. Next, samples were labeled with isobaric tandem mass tags (TMT, 6-plex) and analyzed by liquid chromatography mass spectrometry (Supplementary Table S4). Notably, a peptide corresponding to the transcription factor STAT3 with a phosphorylated tyrosine residue at position 705 was strongly reduced in the SYK inhibitor-treated samples (Fig 6A and Supplementary Fig S16). Tyr705 phosphorylation of STAT3 is required for its dimerization, nuclear translocation and DNA binding (Bromberg *et al*, 1999), indicating that SYK inhibitor-treated cells have reduced activity of STAT3. Inhibition of STAT3 phosphorylation upon SYK inhibition was confirmed by Western blot using a phospho-specific STAT3-Tyr705 antibody (Fig 6B). Finally, we investigated whether STAT3 down-regulation is sufficient to inhibit basal-like breast cancer cell viability. As expected, transduction of the basal-like MDA-MB-468, DU4475 and HCC70 cell lines with a shRNA targeting STAT3 resulted in a pronounced reduction of cell viability (Fig 6C and Supplementary Fig S17). In contrast, knockdown of STAT3 in the luminal cell lines ZR-75-1 and ZR-75-30, which do not respond to PKC412 and SYK inhibitors, also did not show this effect.

B Western blot of MDA-MB-468 cells treated as in (A).

C MDA-MB-468, HCC70, DU4475 (basal) or ZR-75-1, and ZR-75-30 (luminal) cells infected with STAT3 shRNA or non-targeting control shRNA were grown for 4 days, and cell viability was measured via Celltiter Glo. **P < 0.01, ***P < 0.01, two-sided Student's *t*-test. *n* = 3.

D MDA-MB-468 cells treated in dose–response matrix with SYK inhibitor R406 and two different AURKA inhibitors (AT9283 and VX680). Relative cell viability (left) and synergy as expressed by deviation from the Bliss independence model (right) are indicated.

Together, we reveal an unexpected role for the tyrosine kinase SYK in maintaining STAT3 phosphorylation in basal-like breast cancer and provide a rationale for clinically testing SYK small-molecule inhibitors in this subclass of tumors. The mass spectrometry proteomics data have been deposited to the ProteomeXchange Consortium (http://proteomecentral.proteomexchange.org) via the PRIDE partner repository with the dataset identifier PXD001680.

### AURKA and SYK inhibition synergizes in killing basal-like breast cancer cells

Given that AURKA was also bound by PKC412 according to our chemical proteomics data, and depletion in MDA-MB-468 cells abrogated proliferation (Supplementary Fig S8), we investigated whether simultaneous SYK and AURKA inhibition, as presented by PKC412, might show a more than additive effect. Following this polypharmacology model, one may expect that two distinct agents inhibiting these two kinases would result in synergistic impairment of growth of these BLBC cells. Furthermore, the simultaneous inhibition of multiple critical nodes may be more resilient against resistance mechanisms. We therefore performed a dose–response matrix with SYK and AURKA inhibitors and derived synergy scores by calculating the deviation from Bliss additivity (Fig 5D and Materials and Methods). The SYK inhibitor displayed strong synergy with the Aurora inhibitors AT9283 and VX680 across several concentrations. This synergy was not due to R406 inhibiting AURKA or the Aurora inhibitors targeting SYK (Supplementary Figs S18 and S19). This result suggests that simultaneous inhibition of both SYK and AURKA kinases contributes to the mode of action of PKC412. Furthermore, analysis of breast cancer patient data from The Cancer Genome Atlas (TCGA) revealed ubiquitous and elevated expression of both kinases in triple-negative patients—a patient group that is enriched for basal-like breast cancers—indicating that these targets are general co-expressed (Supplementary Fig S20). Together, these data allow us to conclude that multiple targets of PKC412 may act in concert to provide potent and specific activity toward breast cancer cells that display a mesenchymal cell state found in basal-like breast tumors.

## Discussion

The comprehensive profiling of the mutational landscape of TNBC has highlighted only few actionable drug targets in a relatively small fraction of tumors (TCGA, 2012). Therefore, the identification of alternative vulnerabilities in TNBC is critical for the development of novel therapeutic strategies. A subset of TNBCs can be distinguished by their basal-like transcriptional profile, which led us to test the hypothesis whether this specific cell state could be targeted with small molecules. Through a chemical genetic screen and a suite of drug mechanism-of-action studies, we here identify SYK-dependent STAT3 activation as a novel critical vulnerability of basal-like breast cancer cells, next to AURKA, which was previously implicated to be a potential target in BLBC (Staff *et al*, 2010). PKC412 simultaneously inhibits both kinases, causing a synergistic polypharmacology leading to apoptosis.

SYK has been shown to play a role in B-cell leukemia, where it is under investigation in a combination treatment with adjuvant chemotherapy; however, reports on the function of SYK in solid tumors are less clear (Sung *et al*, 2009). Evidence of oncogenic activity of SYK in solid tumors, including breast cancer, has not previously been demonstrated, and neither activating nor inactivating SYK mutations have been identified. Interestingly, SYK has been implicated in the regulation of cell adhesion and migration (Larive *et al*, 2009; Zhang *et al*, 2009), processes related to EMT, and suggested to act as a tumor suppressor in breast cancer (Coopman *et al*, 2000; Sung *et al*, 2009). Together, this suggests that the role of SYK in supporting or suppressing mammary tumors may be highly context dependent and requires further investigation.

A critical role for STAT3 in mammary gland development and tumorigenesis is well documented (Bromberg *et al*, 1999; Chapman *et al*, 1999; Humphreys *et al*, 2002; Dolled-Filhart *et al*, 2003). Elevated STAT3 phosphorylation levels have been reported in basal-like breast cancer cells and are required for breast tumor growth *in vivo* (Buettner *et al*, 2002; Armanious *et al*, 2010; Marotta *et al*, 2011). Furthermore, in human tumors, the highest STAT3 activity is found in CD44^−^/CD24^+^ breast cancer cells, markers associated with tumor-initiating capability, and EMT. Nonetheless, STAT3 activity is observed in other breast cancer subtypes as well, and it is rarely mutated. Several kinases have been implicated in maintaining STAT3 activity in breast cancer, including EGFR, JAK2, and Src (Garcia *et al*, 2001; Marotta *et al*, 2011). Our experiments suggest that in a subset of TNBCs, SYK kinase activity is critically required to maintain STAT3 phosphorylation. Together, this suggests that the upstream regulation of STAT3 in breast cancer cells is complex and that the requirement of individual kinases may be dependent on cell state. This concept is important for the exploitation of STAT3 dependency, as this highlights that different breast cancer subtypes require distinct targeting strategies. Our approach demonstrates that exploiting cell state rather than the mutational landscape of a subset of tumors can lead to the identification of novel treatment strategies.

## Materials and Methods

### Chemicals, genetic screen, and structural clustering

MCF10A cells were infected with a retrovirus encoding mouse Twist1 or control cDNA, and after allowing 14 days for EMT reprogramming, the cells were seeded into 384-well plates and screened against a chemical library of 20,000 chemical compounds including FDA-approved drugs, kinase inhibitors, chromatin modifiers, and a set of structurally diverse compounds (enamine) with unknown activity. The screen was performed in duplicate with 3 days of drug incubation at a concentration of 5–10 μM, depending on the compound. Quantification of viable cells was performed with Celltiter Glo (Promega). The toxic cardiac glycoside sanguinarine (Sigma-Aldrich, Darmstadt, Germany) was used as a positive control and vehicle (DMSO) as a negative control. Celltiter Glo signals were normalized per plate, and hit selection was based on a *z*-score threshold (*z*-score < −2.12) derived from the distribution of positive and negative controls. Jaccard indices were calculated from the structures of the hit compounds and were then clustered using Tanimoto similarity and distance.

PKC412 (Novartis), R406, AB1010 (masitinib), OSI-930, sunitinib, and MLN518 were obtained from Selleckchem. Go6976 and LY333531 were purchased from Tocris Bioscience. BAY61-6306 was obtained from Sigma-Aldrich. For the *in vivo* experiments, PKC412 was kindly provided by Novartis.

### Cell line panel dose–response and synergy analysis

All cell lines were obtained from the ATCC (except MMECs) and cultured in media under conditions as recommended by ATCC. A subset of 28 patient-derived cell lines from the ATCC breast cancer cell line panel was selected based on the similarity of culture conditions and equal representation of basal and luminal subtype. These cells were seeded in 384-well plates and treated with inhibitors in duplicate at 10 different concentrations in a 1:2 dilution series. Cell viability was determined via Celltiter Glo after 3 days of incubation. Results were normalized to DMSO control and displayed as a heatmap. All other dose–response experiments were performed under similar conditions in triplicate in 96-well plates. For synergy experiments, cells were seeded in 96-well plates and drugs were added in triplicate in order to obtain dose–response matrices. Cell viability was quantified using Celltiter Glo after 3 days of incubation. Synergy scores were calculated by determining deviation from Bliss additivity (Bliss, 1939) using the following formula: *E*_*xy*_ = *E*_*x*_ + *E*_*y*_ − (*E*_*x*_*E*_*y*_). Here, *E* is the effect on viability of drugs *x* and *y* expressed as a fraction of the maximum effect.

### Measurement of apoptosis and crystal violet staining

HCC1428 and MDA-MB-468 cells were treated with DMSO, 500 nM camptothecin, or 500 nM PKC412 for 16 h. Then cells were trypsinized, washed, and stained with propidium iodide and annexin V and subjected to FACS (FACSCalibur) analysis to quantify apoptotic fractions.

Cells were grown in 6-well plates until the untreated control reached near confluence. Then, the medium was removed; cells were washed with PBS and fixed using 3.7% paraformaldehyde (PFA). After the removal of PFA, a solution of 0.1% crystal violet in 5% ethanol was added and cells were stained overnight. The next day, staining solution was removed, wells were washed extensively, and images were taken.

### Chemical proteomics and synthesis of 3,4-Bis(3-indolyl)-1*H*-pyrrole-2,5-dion

PKC412 was linked to beads in order to perform affinity chromatography in MDA-MB-468 cell lysates. Pull-downs in lysates were performed with beads only, PKC412-coupled beads (PCB), PCB with the addition of PKC412 (self-competition), and PCB with the addition of LY333531 (competition with non-active compound). Beads were washed, eluted, and treated with trypsin. Peptides corresponding to proteins bound to the beads were identified via mass spectrometry.

3,4-Bis(3-indolyl)-1*H*-pyrrole-2,5-dion was synthesized following a published procedure (Davis *et al*, 1992). Briefly, indole (500 mg, 4.3 mmol) dissolved in benzene (7 ml) was treated with methylmagnesium iodide (1.4 ml of a 3 M solution in diethyl ether), and the resulting solution was stirred at room temperature under an argon atmosphere for 30 min. Dibromomaleimide (300 mg, 1.2 mmol) was added, and the mixture was heated to reflux for 48 h, cooled, and evaporated to dryness. The residue was partitioned between dichloromethane and 2 M HCl. The dichloromethane extract was separated and dried over sodium sulfate, and the combined organic extracts were evaporated to dryness. The resulting residue was subjected to preparative high-pressure liquid chromatography using water 0.1% formic acid/methanol:isopropanol (2:8) as eluant (column: Sunfire Prep.C_18_, 5 μm, 10 × 50 mm, flow: 12 ml/min). After lyophilization, the product could be obtained as red solid in a 30% yield (98 mg, 1.3 mmol). High-pressure liquid chromatography was performed on a Waters 1525 EF binary HPLC pump equipped with a Waters 2998 photodiode array. Mass spectra were recorded on a Waters Xevo TQ system in the electron-positive mode, confirming purity. Lyophilization was performed on a Christ Freeze Dryer Alpha 1-2 LD.

### Western blotting

Cells were treated with compounds for 6 h, and whole-cell lysates were separated on 4–12% SDS–PAGE precast gels (LifeTech). Proteins were transferred to PVDF membranes and subsequently blocked with iBlock (Invitrogen) for 30 min. Then primary antibody was added overnight at 4°C, and after washing with PBS with 1% Tween (PBS-T), secondary HRP-coupled antibody was incubated for 45 min. Blots were once again washed with PBS-T and soaked with ECL substrate (Western Lightning Plus-ECL), and signals were detected with an imaging system (MF-ChemBIS 3.2; DNR Bio-Imaging Systems). Specific proteins were detected using anti-phospho SYK (Cell Signaling Technology #2711) or total SYK protein (Cell Signaling Technology #2712).

### RNA sequencing

MDA-MB-468 cells and ZR75-1 cells were treated with DMSO or PKC412 (1 μM) in triplicate for 6 h. After treatment, RNA was extracted and sequencing libraries for HiSeq2000 were generated using the Truseq 2.0 kit (Illumina). Raw sequencing data were aligned to the human genome, and FPKM values were calculated using a custom analysis pipeline established in-house. Subsequent KEGG pathway enrichment analysis of genes displaying greater than threefold change was performed with the online tool DAVID (http://david.abcc.ncifcrf.gov/). Raw data are accessible at Gene Expression Omnibus (accession number GSE63721).

### Protein–protein interaction network analysis

A network of all known human protein–protein interactions was assembled using available online data from several databases (IntAct, MINT, HPRD, BioGRID, DIP, InnateDB, CORUM). Database versions were the most recent available at the time of generating this integrated network (July 24^th^, 2012). The obtained network was comprised of 13,101 proteins and 127,064 interactions (edges). Basal and luminal-specific networks were obtained by intersecting this generic network with expressed genes (FPKM > 0.1) in the respective cell line RNA-seq data. In basal MDA-MB-468 cells, we found 14,721 genes to be expressed, which resulted in a basal network comprised of 9,096 proteins connected by 103,937 edges after intersection with the generic network. Luminal ZR75-1 cells (14,314 genes expressed) yielded a luminal network comprised of 8,966 proteins linked by 103,733 edges. We devised *ad hoc* edge weights based on mRNA expression fold change measured upon PKC412 versus DMSO treatments in each cell line. We reasoned that the existence of an edge linking two proteins A and B was an indication of concerted activity (Lage *et al*, 2008), and when both proteins were regulated, this augmented the relevance of this interaction in the context of drug treatment. Each edge was hence weighted according to

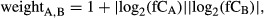
1

where fC_A_ denotes the RNA expression-level fold change of protein A.

Individual drug targets were assigned strengths of interaction with the drug taking the ratio of spectral counts in the immobilized compound versus free compound competition experiments. Using those strengths, we estimated PKC412 treatment impact by means of probabilities resulting from a diffusion process with the targets as seeds. Namely, seeds were assigned initial probabilities summing to 1 and proportional to the interaction strengths. A random walk with restart probability α = 0.3 was applied to diffuse this initial distribution over the network combining its cell line-dependent topology and edge weights. The probabilities assigned to each node of the network were the asymptotic probabilities of the random walk (Colinge *et al*, 2012). Repeating this procedure for 100 randomly chosen targets with identical strengths, we pooled the asymptotic random probabilities and determined the top 5% nodes of the true asymptotic distribution (top 0.5% only represented in Fig 3D for readability). GO terms were scored by setting below 95% node probabilities to 0 (to limit noise) and summing all probabilities over nodes in each GO term. Repeating this procedure for 1,000 random selections of the same number of proteins as annotated in each GO term, we obtained *P*-values for the GO terms. Multiple hypothesis correction (Benjamini–Hochberg) was applied and terms selected at false discovery rate 5% to obtain Supplementary Table S3.

Estimation of the impact of individual PKC412 targets was obtained by recomputing GO term scores as above, one target being removed and the seed probabilities of the remaining ones renormalized to sum up to one; Log_10_ ratios of GO term scores were employed to generate the heatmaps of Fig 3E and Supplementary Fig S7.

### Mouse xenografts

Patient consent for tumor use in animals was obtained under a protocol approved by the Vall d'Hebron Hospital Clinical Investigation Ethical Committee and Animal Use Committee. Tumors were subcutaneously implanted in 6-week-old female HsdCpb:NMRI-Foxn1nu mice (Harlan Laboratories, Italy). After tumor graft growth, tumor tissue was re-implanted into recipient mice, which were randomized upon implant growth.

For the patient-derived basal-like and Cal51 tumor grafts, animals were randomized in 6–8 mice per group when tumor volumes reached 100–200 mm^3^. Mice were treated with PKC412 (100 mg/kg) or vehicle. Tumor grafts were measured with calipers, and tumor volumes were determined using the formula: (length × width^2^) × (π/6). At the end of the experiment, animals were sacrificed by CO_2_ inhalation. Tumor volumes are plotted as mean ± SE.

Difference between PKC412-treated and vehicle-treated tumors was determined by calculating the Akaike information criterion (AIC) statistics in GraphPad Prism 5.

### Molecular docking

The X-ray structure of SYK co-crystallized with staurosporine (PDB code: 1XBC; Atwell *et al*, 2004) was downloaded from the RCSB protein data bank (Bernstein *et al*, 1977) and prepared using the pdb2 receptor tool of Open Eye's OEDocking suite (Ref. OEDocking version 3.0.1. OpenEye Scientific Software, Santa Fe, NM, http://www.eyesopen.com). Potential 3D conformations of the ligands, staurosporine aglycon and its flexible derivative, were calculated using the program OMEGA (Hawkins *et al*, 2010; Hawkins & Nicholls, 2012) (Ref. OMEGA version 2.5.1.4. OpenEye Scientific Software, Santa Fe, NM, http://www.eyesopen.com) and stored in a multi-conformer database. Because of the availability of the co-crystal structure of SYK with staurosporine, the docking program HYBRID, implemented in the OEDocking suite, was selected for the molecular docking study. This tool takes advantage of bound ligand information for pose prediction, which results in improved docking results (McGaughey *et al*, 2007). Choosing the highest docking resolution, the precalculated ligand conformations were docked sequentially into the prepared protein structure, generating 100 docking poses per ligand.

As we assumed the binding mode of the aglycon to be analogous to the one of staurosporine, the poses were prefiltered for the interactions known to be important for staurosporine binding (H-bonds to Ala451 and Glu449, depicted in Fig 4A). By that, ligand poses showing wrong orientations in the binding site could be ruled out. The scoring function Chemgauss4 was used for estimating the binding energies of the resultant binding hypotheses.

### *In vitro* Syk kinase assay

*In vitro* kinase activity was measured using the SYK Kinase Enzyme System (Promega). Recombinant SYK protein was incubated with vehicle (DMSO), PKC412 (250 nM), staurosporine aglycon (250 nM), staurosporine aglycon derivative (250 nM), or the SYK inhibitor R406 (250 nM) according to the manufacturer's protocol, and kinase activity was measured using a luminescence reader (Victor 3; Perkin Elmer).

### Lentivirally mediated depletion and overexpression of SYK cDNA

Targeting shRNA sequences for STAT3 (targeting sequence: GCACAATCTACGAAGAATCAA), SYK (1:GCAGGCCATCATCAGTCAGAA, 2:CGACAAAGACAAGACAGGGAA), and FER (1:CAAACATTCCTCAACTTATAG, 2:CAGAACAACTTAGTAGGATAA) were obtained from the TRCN database (http://www.broadinstitute.org/rnai/public/gene/search) and cloned into the lentiviral vector pLKO.1 using AgeI and EcoRI restriction sites. Identity of cloned vectors was verified by sequencing, and lentiviral particles were produced by calcium phosphate transfection of the vectors along with the packaging plasmids into HEK293T cells. GFP was used as a transfection control. Two days after transfection, virus-containing supernatant was harvested, centrifuged at approximately 140 *g* for 10 min, and filtered to remove HEK293T cells from the supernatant. Target cells were infected with the virus-containing supernatant in the presence of polybrene (final concentration 8 μg/ml). Infected cells were selected using puromycin (2 μg/ml; Sigma-Aldrich). For overexpression/rescue experiments, a SYK cDNA in a retroviral vector was obtained from Addgene (pWZL Neo Myr Flag SYK) and retroviral supernatant was produced analogous to the lentiviral virus production only substituting the polymerase packaging vector for a retroviral one.

### Quantitative liquid chromatography/mass spectrometry analysis of phosphotyrosine-enriched peptides

Total cell extract was prepared by lysing 1× PBS-washed cells in 5 ml 8 M urea lysis buffer containing PhosSTOP phosphatase and complete protease inhibitor cocktails (Roche Diagnostics GmbH, Mannheim, Germany). 5 × 15 cm culture dishes were used per condition. Cell extraction and DNA shearing was assisted by sonication (S2X; Covaris Inc) and cell debris pelleted by centrifugation at 20,000 *g* for 5 min at 4°C. The supernatant was removed and protein concentration determined using the 660 nm protein assay (Pierce; Thermo Fisher Scientific, Waltham, MA, USA). A total of 6 mg protein per condition was reduced with DTT (10 mM), alkylated with iodoacetamide (55 mM), and digested with modified porcine trypsin (1:100; Promega Corp., Madison, WI, USA) at 37°C for approximately 24 h. Quenched peptide digest was extracted by solid-phase extraction using Sep-Pak classic C18 cartridges (Waters Corporation, Milford, MA, USA). The eluate was lyophilized and peptides resuspended in 100 mM Tris–HCl, pH 7.4, containing 0.3% NP-40 and phosphotyrosine-containing peptides enriched by immuno-precipitation using equal amounts of the anti-phosphotyrosine antibodies PT66 (Sigma-Aldrich GmbH), P-Tyr-100 (Cell Signaling Technologies Inc, Danvers, MA, USA), and 4G10 (Millipore, Billerica, MA, USA) conjugated to G Sepharose beads (GE Healthcare, Uppsala, Sweden). The immunoprecipitation was performed for 12 h, the beads washed, and the peptides eluted with 5% formic acid. After lyophilization to remove formic acid, peptides were labeled with TMT 6-plex reagents (Pierce; Thermo Fisher Scientific Inc) as follows: DMSO control (TMT labels 126 and 129); inhibitor R406, 250 nM (TMT labels 127 and 130); and inhibitor BAY61-3606 (TMT labels 128 and 131), 250 nM. The subsequently pooled sample was additionally enriched for phosphotyrosine-containing peptides using a modified IMAC procedure (Ficarro *et al*, 2009). The final sample was analyzed as a single injection on a hybrid linear trap quadrupole (LTQ) Orbitrap Velos mass spectrometer (Thermo Fisher Scientific) using the Xcalibur version 2.1.0.1140 coupled to an Agilent 1200 HPLC nanoflow system via a nano-electrospray ion source using liquid junction (Proxeon, Odense, Denmark). The analyses were performed in a data-dependent acquisition mode using a top 10 hybrid method with a collision-induced dissociation (CID) MS^2^ event followed by a high-energy C-trap dissociation (HCD) MS^2^ event for each selected precursor ion. Acquired RAW data were analyzed by the Proteome Discoverer 1.4.0.288 platform integrating MASCOT, phosphoRS, and target decoy PSM validator software modules. Database search was performed against the human SwissProt database (sp_human_v2013.1) with the following parameters: fixed modification of carbamidomethylation (C); variable modification of oxidation (M) and phosphorylation (S, T and Y); two miscleavage sites; search tolerance of 10 ppm on precursor; and 0.6 or 0.1 Da on fragment ions for CID and HCD, respectively. Actual strict false discovery rate was calculated to be below 1%. Relative quantitation was calculated by taking the ratios of TMT reporter ion intensities. Identification and quantitation were assessed manually for selected phosphopeptides.

## Supporting Information

Supplementary Figure and Table Legends

Review Process File
