# Supplementary figures and images for: Targeting a cell state common to triple-negative breast cancers
Source: Mol Syst Biol. 2015 Feb 19;11(2):789. doi: 10.15252/msb.20145664 (PMC4358660; doi:10.15252/msb.20145664)

Supplementary Figure 1

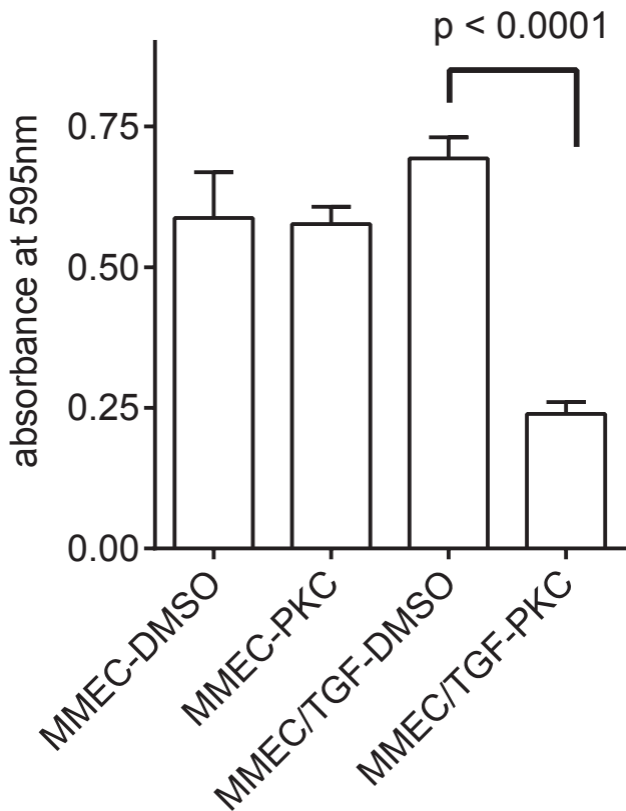

Supplement: Supplementary file 1 [file msb0011-0789-sd1.pdf]

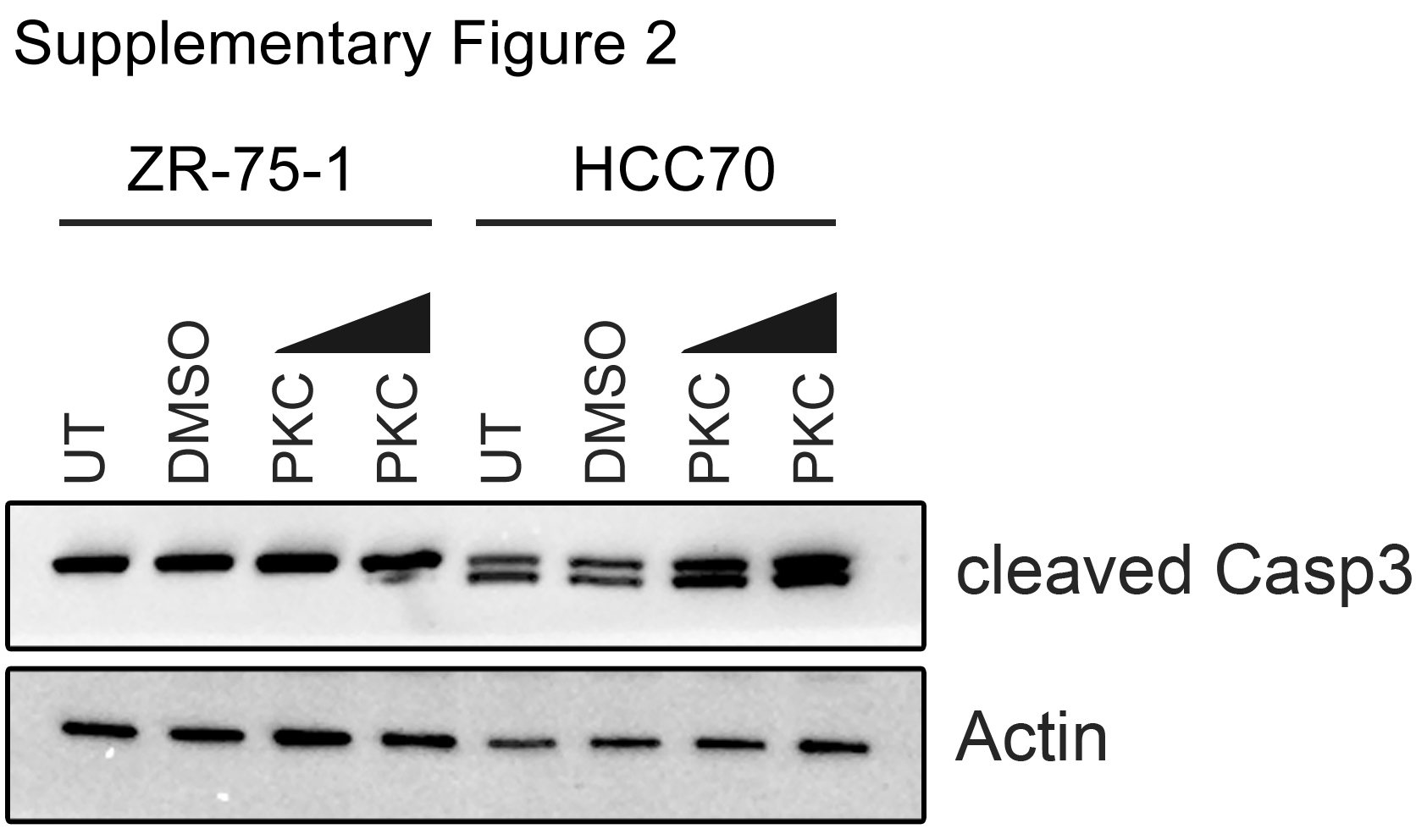

Supplement: Supplementary file 2 [file msb0011-0789-sd2.jpg]

# Supplementary Figure 3

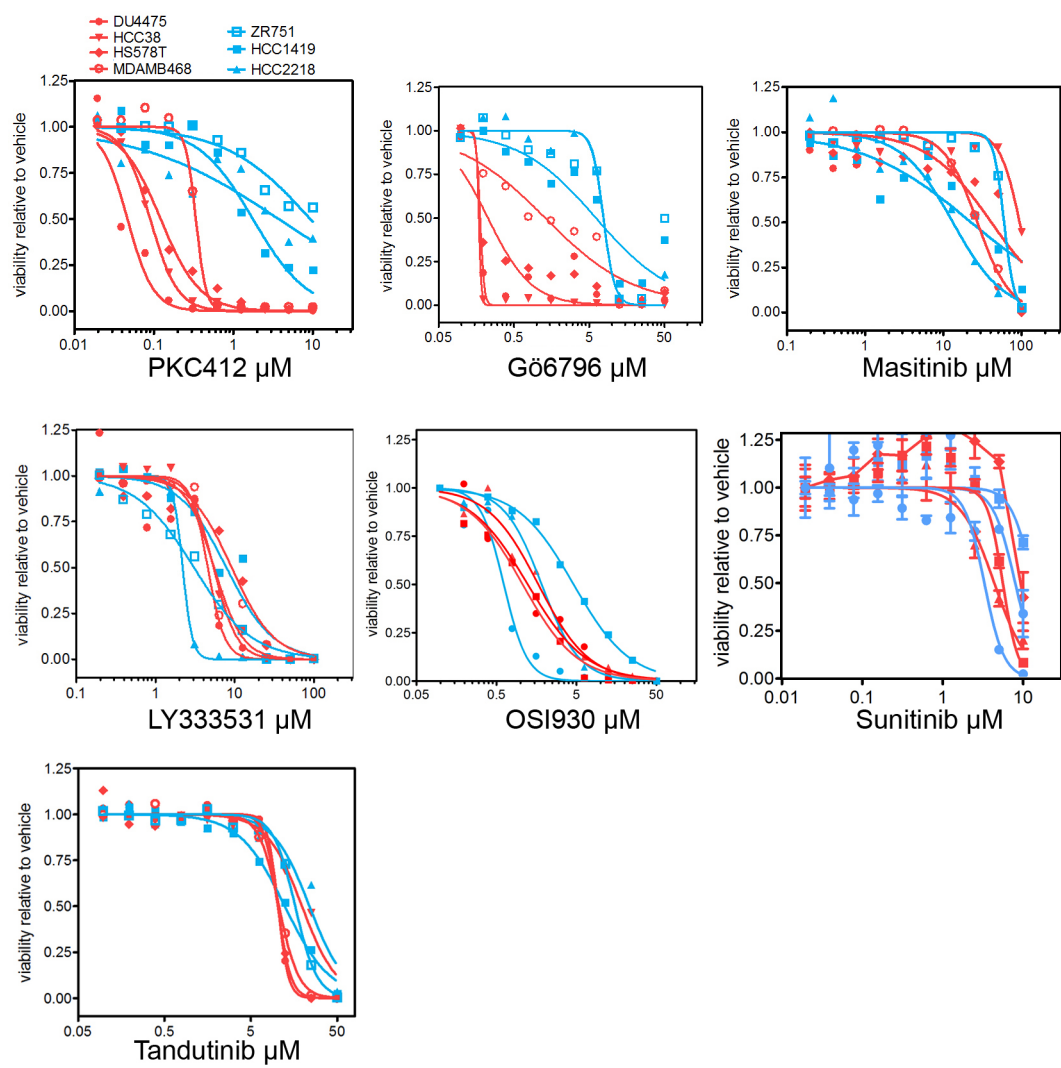

Supplement: Supplementary file 3 [file msb0011-0789-sd3.pdf]

Supplementary Figure 4

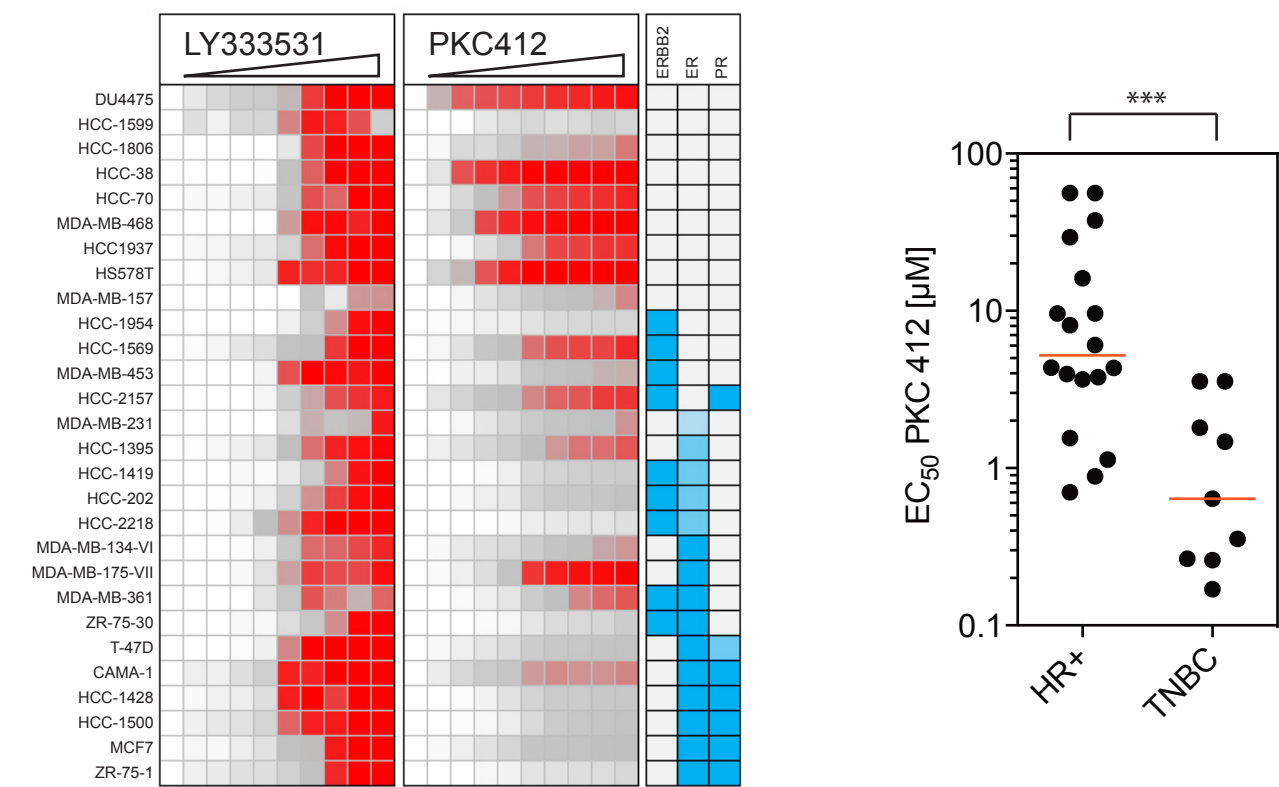

Supplement: Supplementary file 4 [file msb0011-0789-sd4.pdf]

Supplementary Figure 6

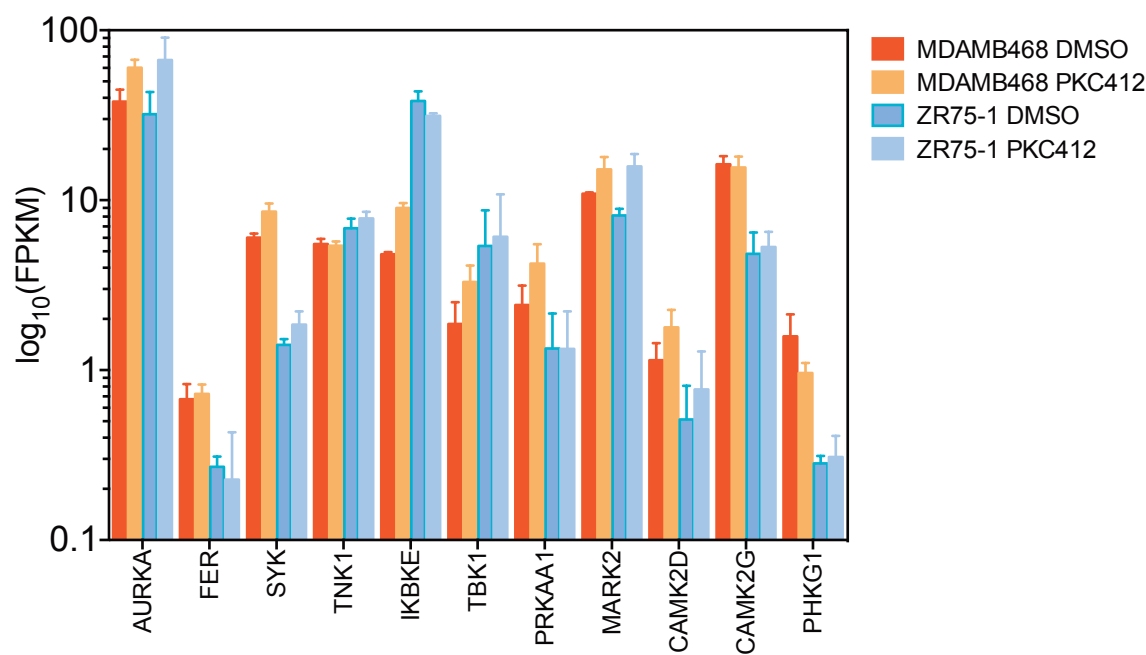

Supplement: Supplementary file 6 [file msb0011-0789-sd6.pdf]

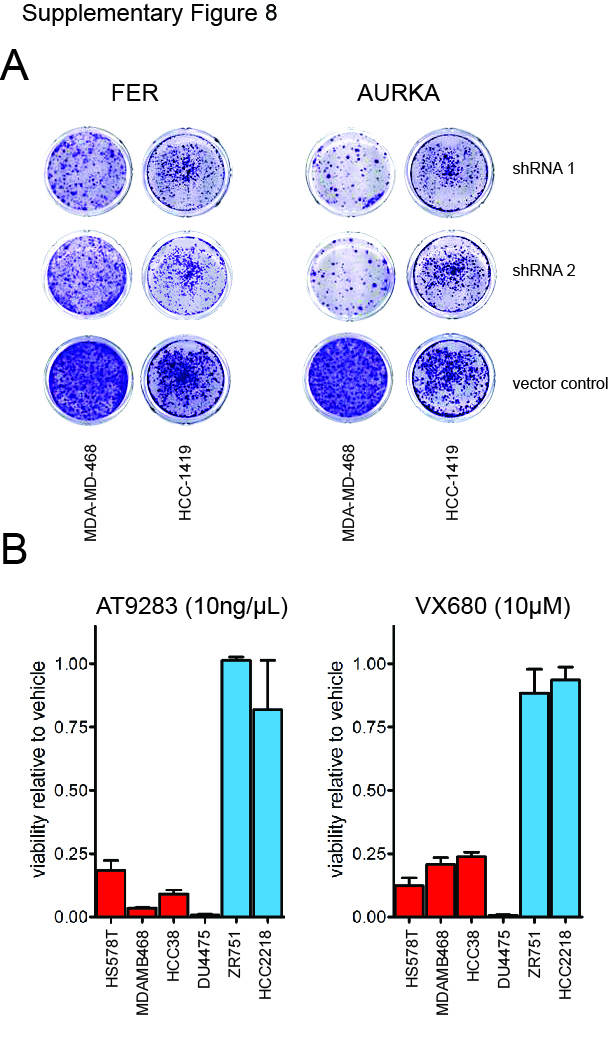

Supplement: Supplementary file 8 [file msb0011-0789-sd8.jpg]

Supplementary Figure 9

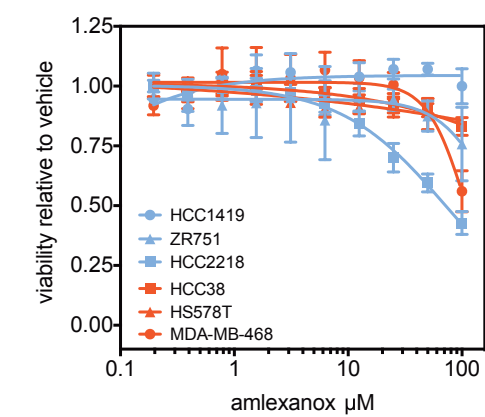

Supplement: Supplementary file 9 [file msb0011-0789-sd9.pdf]

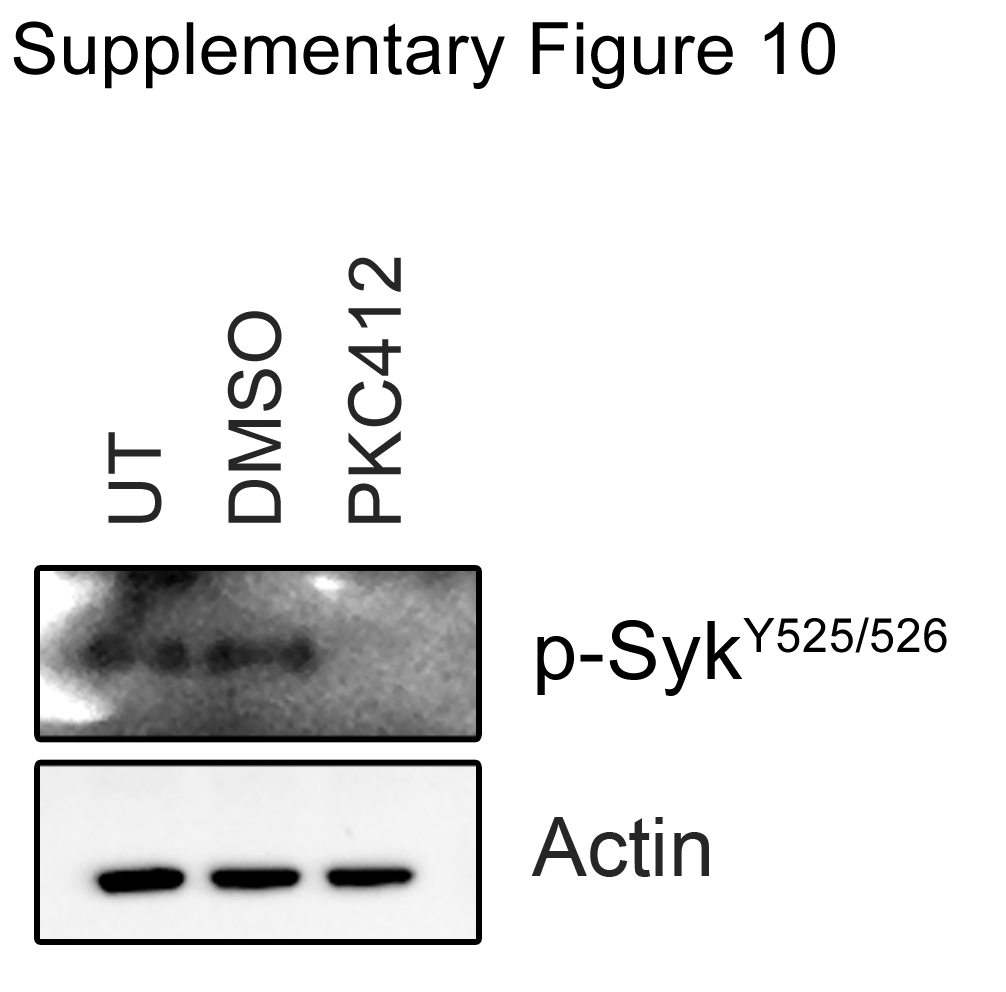

Supplement: Supplementary file 10 [file msb0011-0789-sd10.jpg]

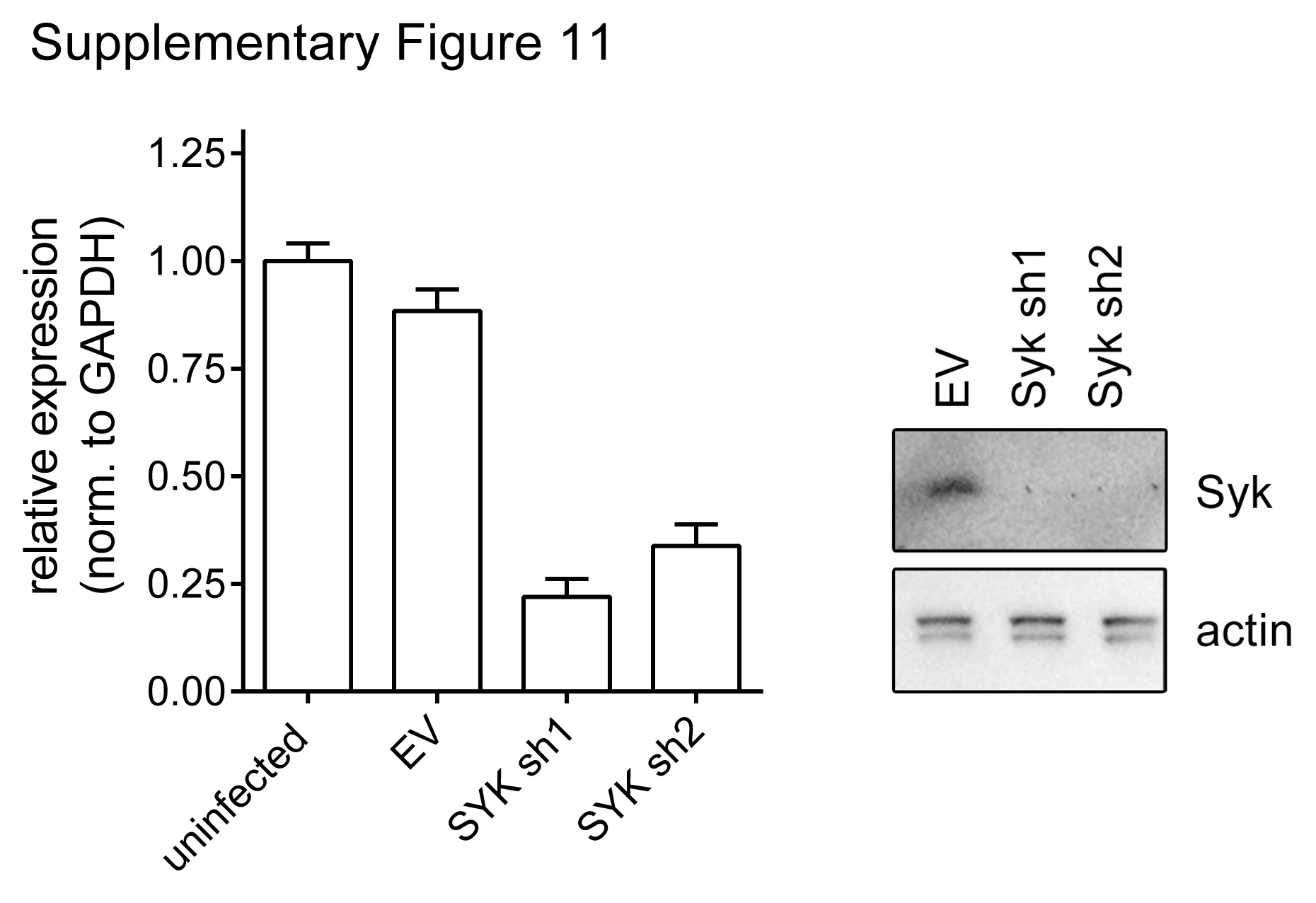

Supplement: Supplementary file 11 [file msb0011-0789-sd11.jpg]

Supplementary Figure 12

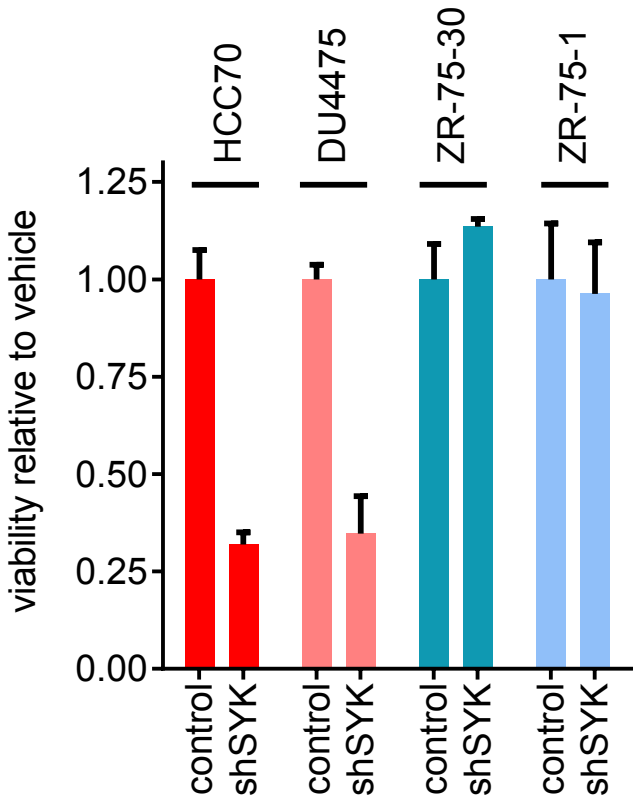

Supplement: Supplementary file 12 [file msb0011-0789-sd12.pdf]

# Supplementary Figure 13

20130417\_BEH(FA\_ACN)

20130417\_CT001 144 (1.060)

1: MS2 ES+  
4.31e7

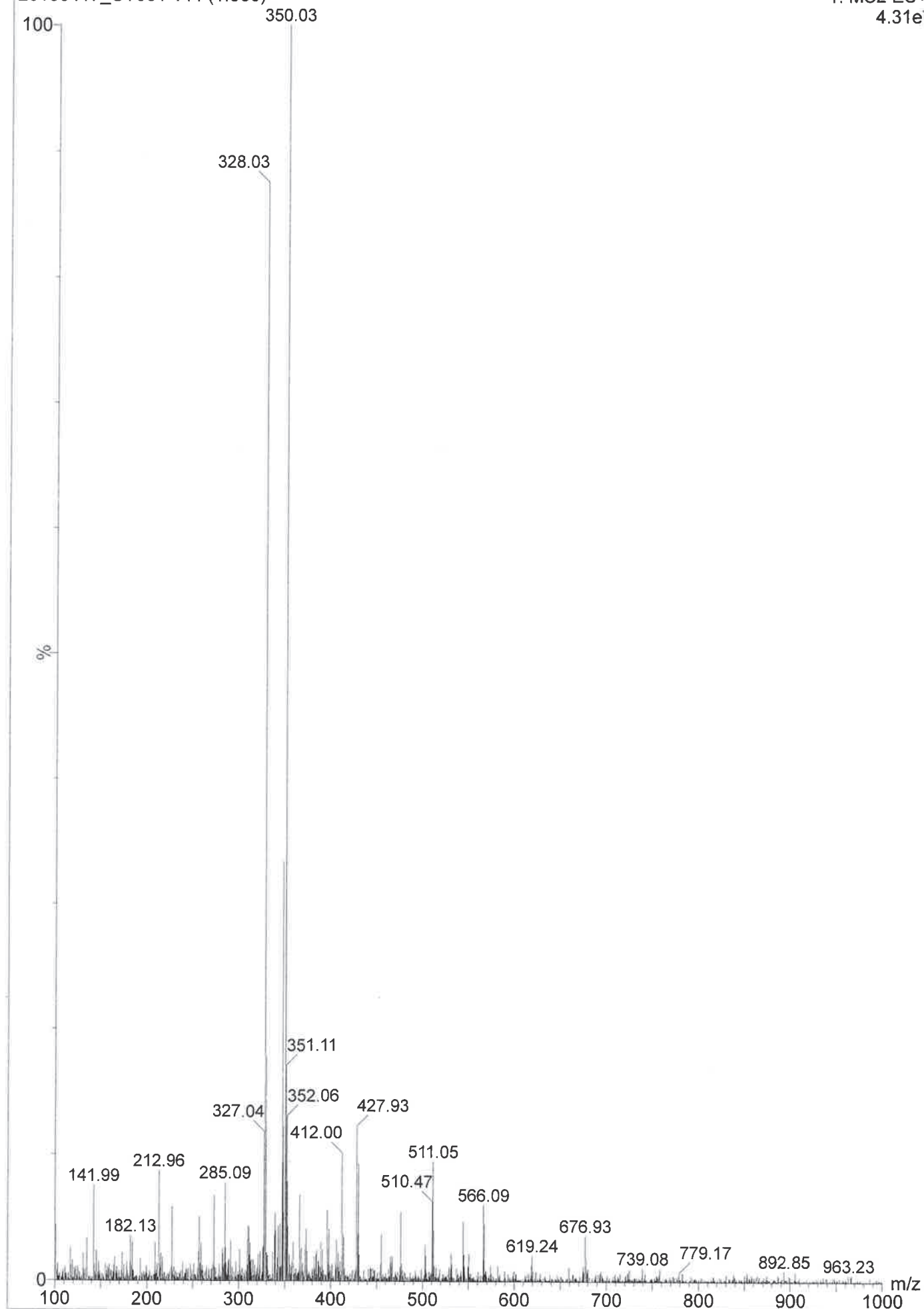

Supplement: Supplementary file 13 [file msb0011-0789-sd13.pdf]

Supplementary Figure 14

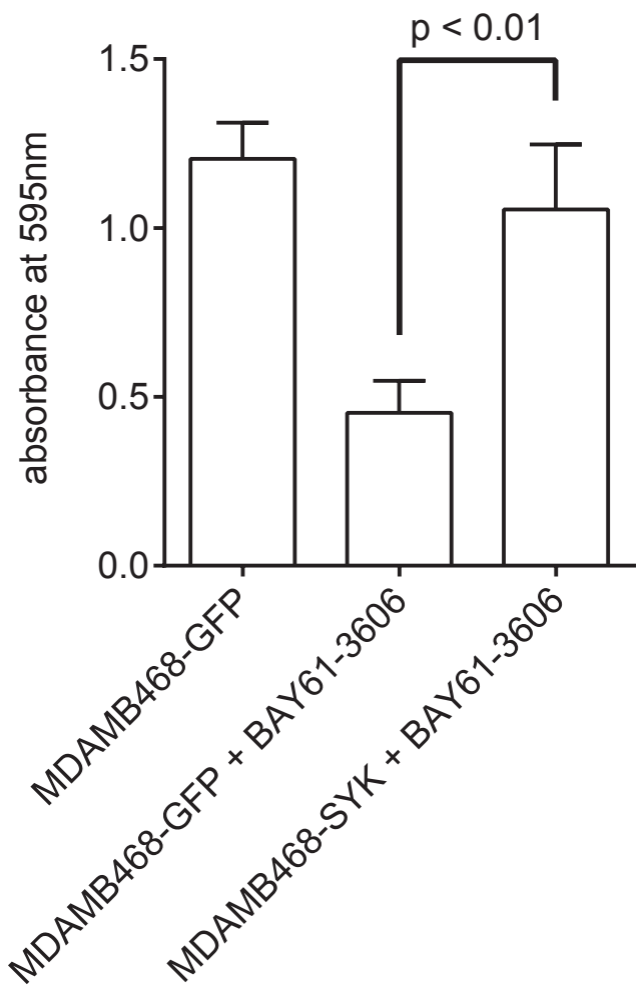

Supplement: Supplementary file 14 [file msb0011-0789-sd14.pdf]

Supplementary Figure 15

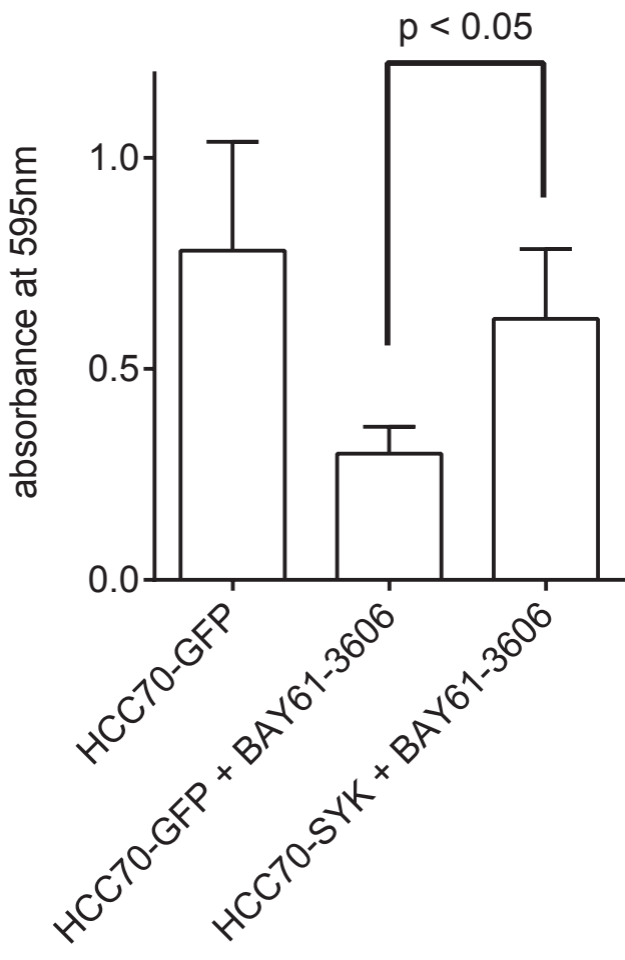

Supplement: Supplementary file 15 [file msb0011-0789-sd15.pdf]

Supplementary Figure 16

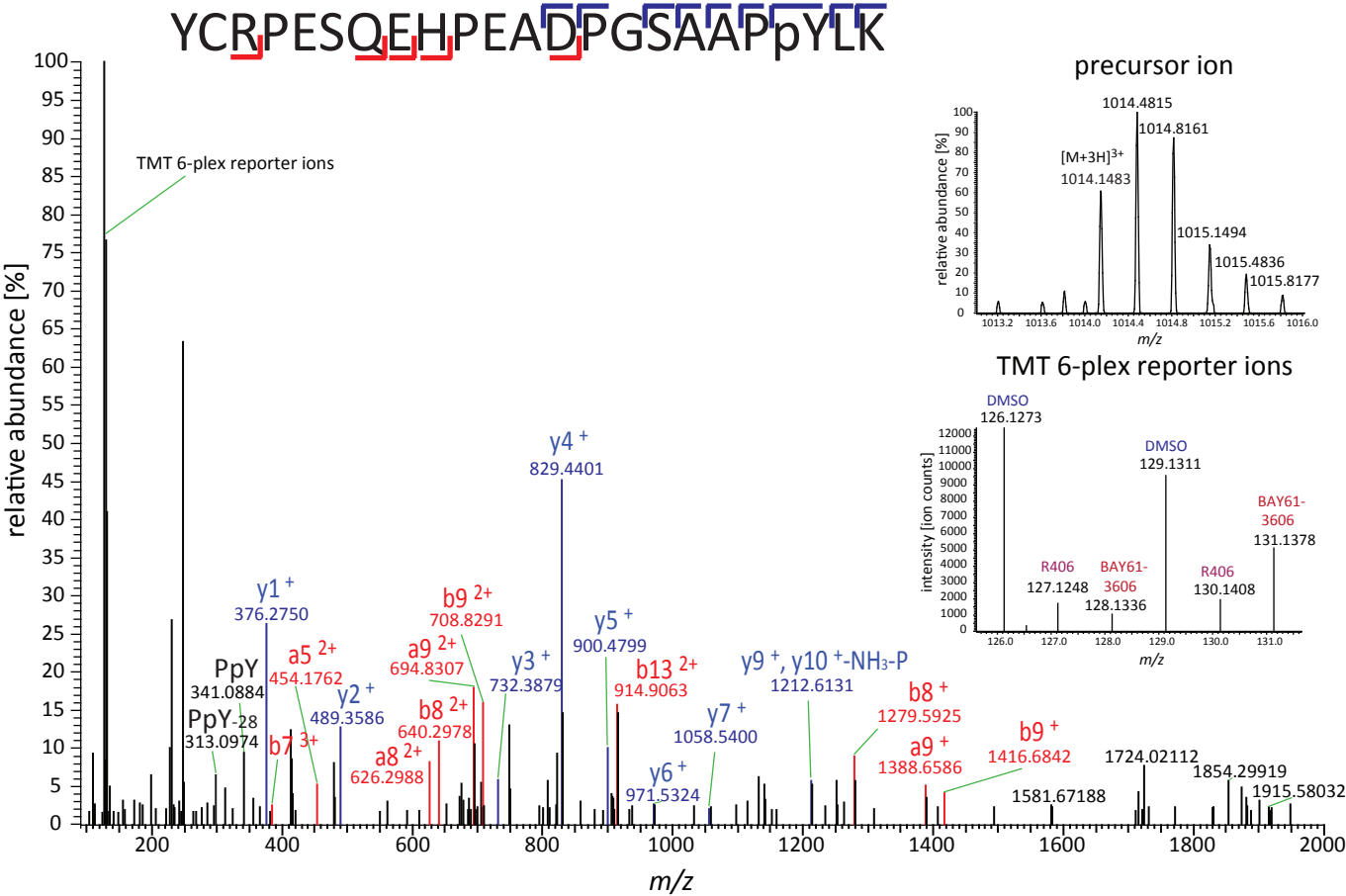

Supplement: Supplementary file 16 [file msb0011-0789-sd16.pdf]

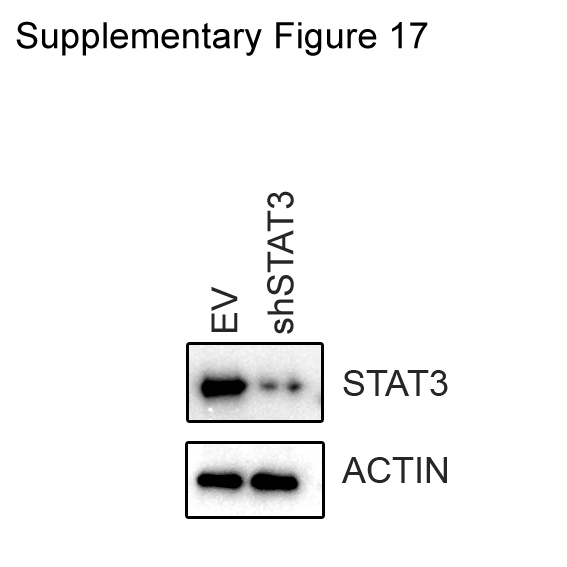

Supplement: Supplementary file 17 [file msb0011-0789-sd17.jpg]

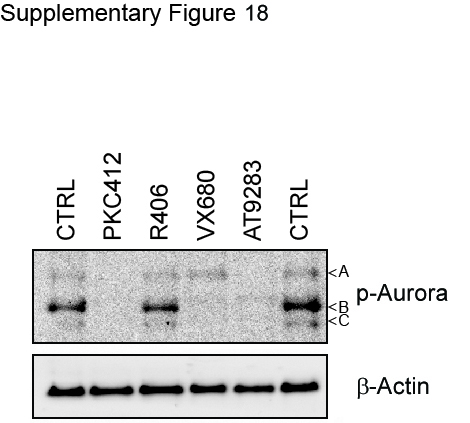

Supplement: Supplementary file 18 [file msb0011-0789-sd18.jpg]

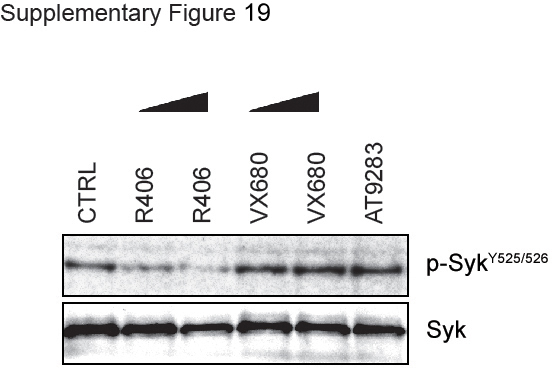

Supplement: Supplementary file 19 [file msb0011-0789-sd19.jpg]

Supplementary Figure 20

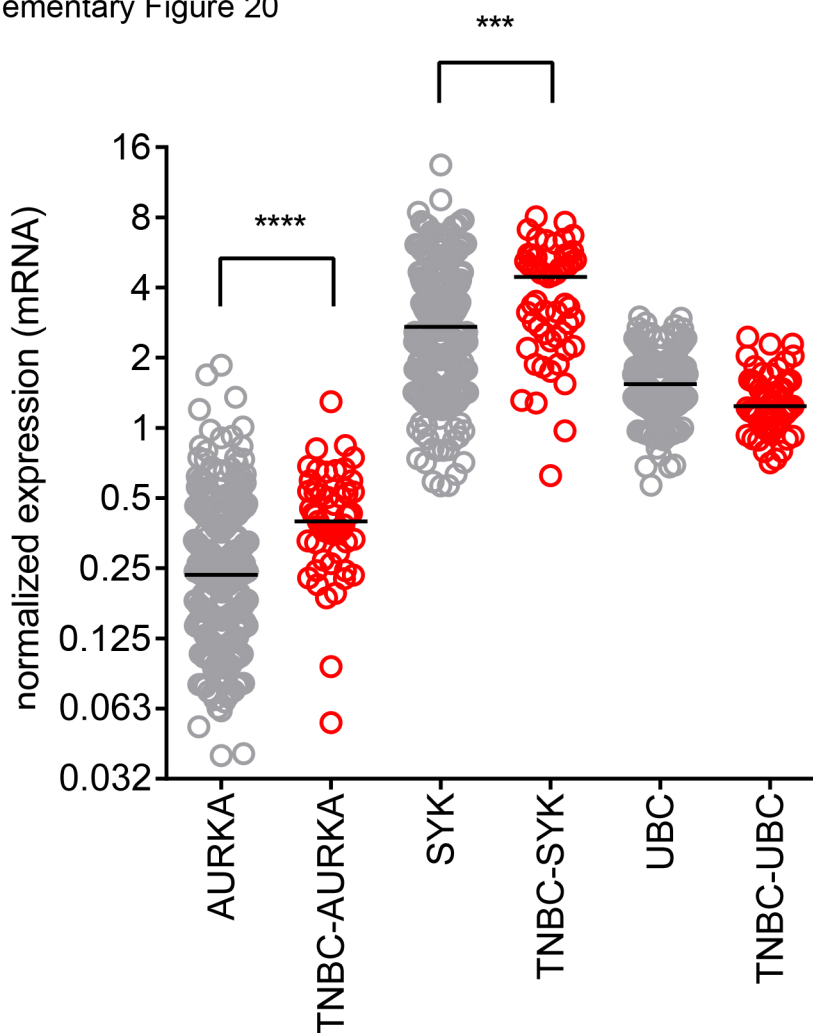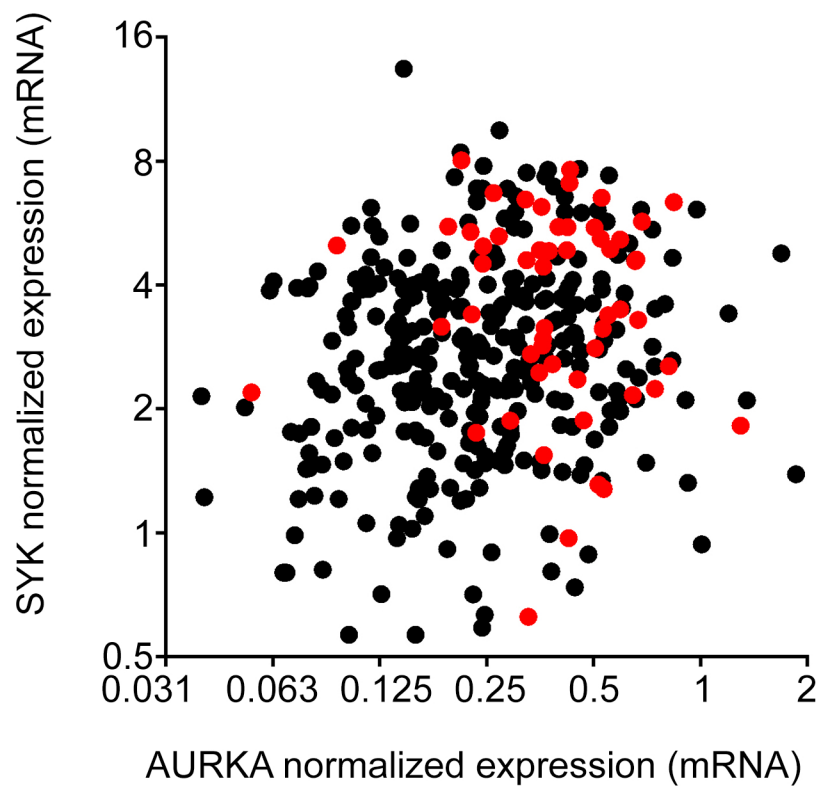

Supplement: Supplementary file 20 [file msb0011-0789-sd20.pdf]
