# Supplementary material for: Targeting a cell state common to triple-negative breast cancers
Source: Mol Syst Biol. 2015 Feb 19;11(2):789. doi: 10.15252/msb.20145664 (PMC4358660; doi:10.15252/msb.20145664)
Supplement: Supplementary file 5 [file msb0011-0789-sd5.pdf]

Supplementary Figure 5

enriched pathways in MDA-MB-468 (basal) after treatment with PKC412			
Category	Term	P-Value	Benjamini
KEGG_PATHWAY	Cytokine-cytokine receptor interaction	6.20E-04	8.10E-02
KEGG_PATHWAY	Apoptosis	9.60E-04	6.40E-02
KEGG_PATHWAY	NOD-like receptor signaling pathway	9.40E-03	3.50E-01
KEGG_PATHWAY	Small cell lung cancer	1.10E-02	3.30E-01
KEGG_PATHWAY	Axon guidance	1.30E-02	3.10E-01
KEGG_PATHWAY	Chemokine signaling pathway	2.20E-02	3.90E-01
KEGG_PATHWAY	Pathways in cancer	3.10E-02	4.60E-01
KEGG_PATHWAY	TGF-beta signaling pathway	4.30E-02	5.30E-01
KEGG_PATHWAY	p53 signaling pathway	4.90E-02	5.40E-01
KEGG_PATHWAY	Regulation of actin cytoskeleton	5.20E-02	5.20E-01
KEGG_PATHWAY	Focal adhesion	7.20E-02	6.10E-01
KEGG_PATHWAY	Cell cycle	7.50E-02	5.90E-01
KEGG_PATHWAY	Jak-STAT signaling pathway	8.60E-02	6.10E-01
KEGG_PATHWAY	Pathogenic Escherichia coli infection	8.60E-02	5.90E-01
KEGG_PATHWAY	MAPK signaling pathway	9.40E-02	5.90E-01

enriched pathways in ZR-75-1 (luminal) after treatment with PKC412			
Category	Term	P-Value	Benjamini
KEGG_PATHWAY	Colorectal cancer	3.10E-03	2.80E-01
KEGG_PATHWAY	Neuroactive ligand-receptor interaction	1.10E-02	4.30E-01
KEGG_PATHWAY	GnRH signaling pathway	2.90E-02	6.50E-01
KEGG_PATHWAY	Dorso-ventral axis formation	3.60E-02	6.10E-01
KEGG_PATHWAY	Pancreatic cancer	5.50E-02	6.90E-01
KEGG_PATHWAY	Phosphatidylinositol signaling system	5.80E-02	6.50E-01
