# Supplementary material for: Targeting a cell state common to triple-negative breast cancers
Source: Mol Syst Biol. 2015 Feb 19;11(2):789. doi: 10.15252/msb.20145664 (PMC4358660; doi:10.15252/msb.20145664)

Supplementary Figure 7

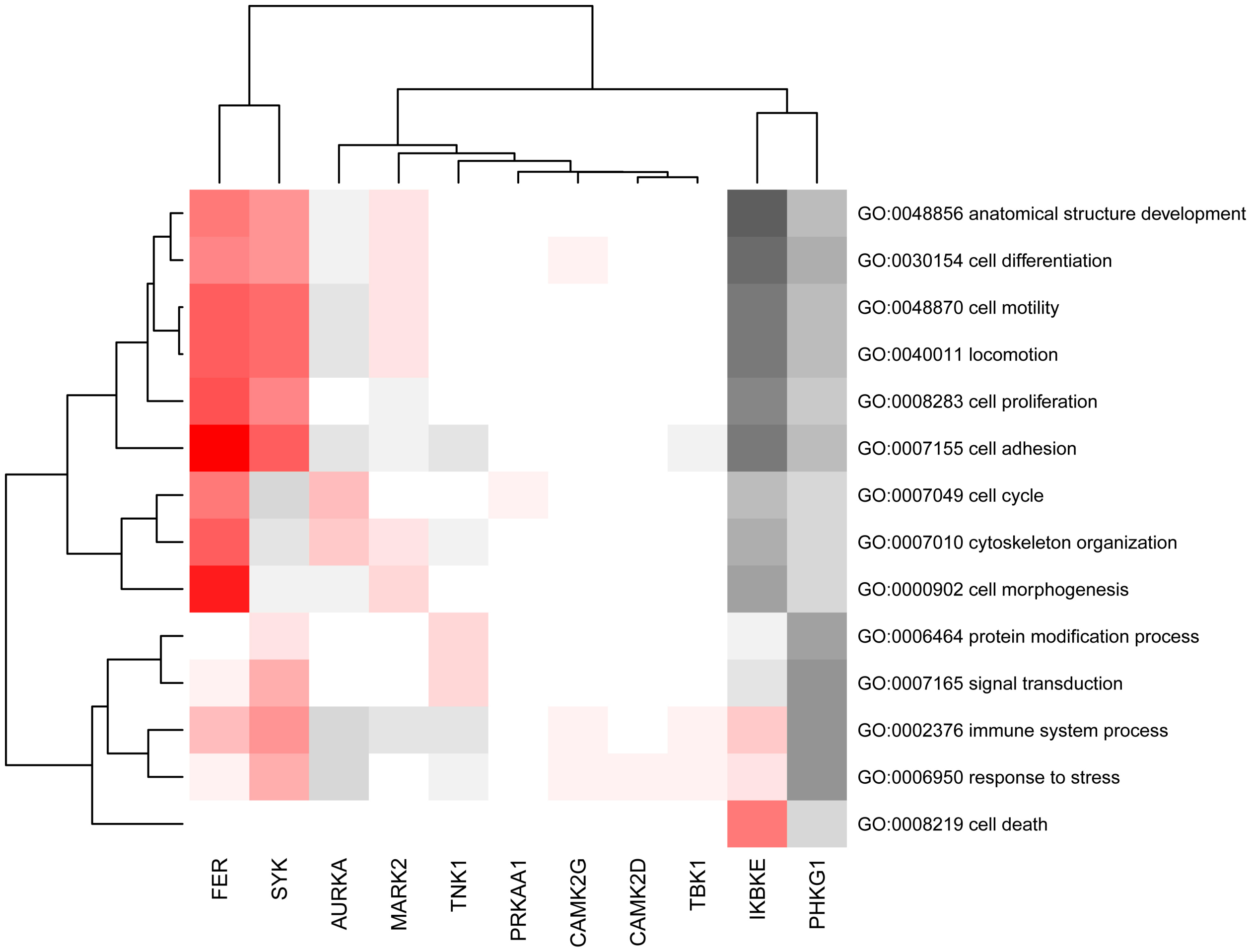

Supplement: Supplementary file 7 [file msb0011-0789-sd7.pdf]
