# Supplementary material for: Targeting a cell state common to triple-negative breast cancers
Source: Mol Syst Biol. 2015 Feb 19;11(2):789. doi: 10.15252/msb.20145664 (PMC4358660; doi:10.15252/msb.20145664)
Supplement: Supplementary file 27 [file msb0011-0789-sd27.docx]

**Supplementary Figure and Table Legends**

**Figure S1 | Quantification of MMEC-Ras^V12^ cells pre-treated with TGF and incubated with PKC412**

MMEC-HRAS^V12G^ cells treated with TGF-β (10ng/µL) or vehicle for 14 days to induce a mesenchymal phenotype. Then cells were treated with PKC412 (500nM) for 7 days and stained with crystal violet. Stains were quantified by photometry at 595nm after extraction with 50% ethanol/1% acetic acid. (mean ± sd; n = 3; t-test two sided)

**Figure S2 | PKC412 induces apoptosis in HCC70 but not ZR-75-1 cells**

Basal HCC70 and luminal ZR-75-1 cells were treated with DMSO, 250nM and 500nM PKC412 or left untreated for 16h and were harvested for western blot. An increase in cleaved caspase3 (Cell Signaling #9661S) was seen in HCC70 but not ZR-75-1 cells.

**Figure S3 | Canonical PKC412 target analysis on breast cancer cell line panel.**

Dose-response curves for indicated compounds inhibiting canonical PKC412 targets on selected basal-like (red lines) and luminal (blue lines). Indicated is the mean viability (n=3) for each dose point after 96 hour compound treatment. Shown is a representative experiment performed at least three times. (mean, curve fit using "sigmoidal fit" model in graphpad prism, n >= 3).

**Figure S4 | PKC412 selectively kills triple negative breast cancer cells.**

The indicated cell lines were treated with PKC412 or the non-selective analog LY333531 in dose-response. Cell lines are sorted by the expression of HER2, ER and PR (blue boxes). Cell viability (celltiter glo) is displayed and white to red shading indicates decreasing viability. (mean, n = 2 for each data point obtained, Mann-Whitney test)

**Figure S5 | Transcriptome and KEGG pathway analysis after PKC412 treatment.**

Transcriptomes of basal-like (MDA-MB-468) or luminal (ZR-75-1) cells treated with PKC412 (500nM, 6 hours) or DMSO was determined using RNA-seq. KEGG pathway enrichment was calculated with the DAVID online tool for all genes up or downregulated more than 3 fold. P values and false discovery rates (Benjamini score) are indicated.

**Figure S6 | Expression of PKC412 targets in breast cancer cells.**

Expression levels (FPKM) for each of the eleven candidate PKC412 targets in basal-like (MDA-MB-468) or luminal (ZR-75-1) cell lines upon DMSO or 500 nM PKC412 for 6 hours. (mean ± sd, n = 3)

**Figure S7 | Gene Ontology heatmap of in silico protein-protein network perturbations.**

Heatmap showing the impact of the individual PKC412 targets on the indicated GO-terms. Red represents reduced association with the GO term, therefore indicating potentially essential targets. Grey represents dispensable targets.

**Figure S8 | Knockdown of FER in breast cancer cell lines.**

(**a**) Basal MDA-MB-468 or luminal HCC-1419 cells were infected with two independent hairpins for FER, AURKA or vector controls. Cells were stained with crystal violet 6 days after infection. (**b**) Basal (red) or luminal (blue) cell lines were treated with AURKA inhibitors VX680 or AT9283 at the indicated concentrations and cell viability was measured with celltiter-Glo after 3 days. (mean ± sd, n=3)

**Figure S9 | Chemical IKBKE inhibition in breast cancer cell lines.**

Three basal (red) and three luminal (blue) cells were treated with the IKBKE inhibitor Amlexanox for 3 days. Cell viability was measured with celltiter glo. Shown is a representative experiment (mean ± sd, curve fit using "sigmoidal fit" model in graphpad prism, n=3).

**Figure S10 | Western blot for p-Syk^Y525/526^ after PKC412 treatment**

MDA-MB-468 cells were treated with PKC412 at 1µM for 8h, lysed and probed with anti-phospho Syk^Y525/526^ or Actin.

**Figure S11 | Knockdown of Syk via short hairpin RNA**

MDA-MB-468 cells were infected with a vector encoding shRNAs against SYK, empty vector or left uninfected. qRT PCR (bar graphs) and a western blot was performed 48h after infection to verify efficient knockdown. (mean ± ds, n = 3)

**Figure S12 | Knockdown of SYK phenocopies Syk kinase inhibitor treatment**

HCC70, DU4475 (basal) or ZR-75-1 and ZR-75-30 (luminal) cells were infected with SYK shRNA 1 or non-targeting control shRNA, were grown for 4 days and cell viability was measured via Celltiter Glo. Basal cell lines were significantly more sensitive (mean ± sd, n = 3, students t-test two sided, p < 0.001 in both cases, luminal n.s.).

**Figure S13 | MS spectrum of the synthesized staurosporin aglycon derivative**

HPLC-MS spectra were recorded in the electron-positive mode to confirm purity.

**Figure S14** **| Overexpression of SYK rescues MDA-MB-468 sensitivity to Syk inhibition.**

MDA-MB-468 cells transfected with a SYK cDNA or GFP expressing vector were treated with 10µM BAY61-3606 for 7 days. Cells were stained using crystal violet. Photometric quantification at 595nm was performed after extraction with 50% ethanol/1% acetic acid. (mean ± sd, n = 3, students t-test two sided)

**Figure S15** **| Overexpression of SYK rescues HCC70 sensitivity to Syk inhibition.**

HCC70 cells transfected with a SYK cDNA or GFP expressing vector were treated with 10µM BAY61-3606 for 7 days. Cells were stained using crystal violet. Photometric quantification at 595nm was performed after extraction with 50% ethanol/1% acetic acid. (mean ± ds, n = 3, students t-test two sided)

**Figure S16** **| Raw MS data for the identification of the STAT3^Y705^ site.**

Peptide fragmentation sites are indicated.

**Figure S17** **| Western blot showing SYK and AURKA inhibitors on p-aurora**

MDA-MB-468 cells were treated with indicated drugs at 10µM concentration for 16h, lysed and probed with anti-phospho AURKA(Thr288)/B(Thr232)/C(Thr198) antibody (Cell Signaling #2914) or anti-actin.

**Figure S18** **| Western blot showing knockdown of STAT3**

MDA-MB-468 cells were infected with a vector encoding shRNAs against STAT3 or empty vector. Western blot was performed 48h after infection to verify efficient knockdown.

**Figure S19** **| Western blot showing SYK and AURKA inhibitors on p-SYK^Y525/526^**

MDA-MB-468 cells were treated with indicated drugs at 1µM and 10µM for 16h, lysed and probed with anti-phospho SYK^Y525/526^ or SYK.

**Figure S20** **| TCGA Data shows that both SYK and AURKA are expressed in TNBCs**

Breast cancer data from the cancer genome atlas (http://cancergenome.nih.gov/) was downloaded on 25.09.2014. The initial 2,205 cases were filtered by availability of transciptomics data and status information on ER, PR and HER2. 376 cases remained which were split into triple negative (TNBC, no expression of ER and PR and no overexpression of HER2; n = 55) or non-triple negative cases (n = 321). mRNA Expression of Aurora Kinase A, Spleen Tyrosine Kinase and Ubiquitin C (used as a positive control for expression). Mann-Whitney U-test. Bottom panel: scatter plot between AURKA and SYK mRNA expression. TNBC are indicated as red dots.

**Table S1 | Chemical genetic screen results.**

MCF10A-Twist1 and control MCF10A cell line specific cytotoxic compounds from the screen. Z-scores are indicated.

**Table S2** **| PKC412 chemical proteomics.**

Peptide counts from chemical proteomics pulldowns in MDA-MB-468 cell lysate with immobilized PKC412 either uncompeted or competed with PKC412 itself or the unselective structural analog LY333531. Peptide counts per protein for each replicate are indicated.

**Table S3 | Gene Ontology analysis of protein-protein interaction network around the PKC412 chemical proteomics targets.**

Indicated are all significantly enriched GO biological processes.

**Table S4 | Phosphoproteomics of SYK inhibitor treated MDA-MB-468 cells.**

Results from the tyrosine phosphoproteomics experiment using two different SYK inhibitors or vehicle (DMSO) on MDAMB468 cells
