# Supplementary material for: Targeting a cell state common to triple-negative breast cancers
Source: Mol Syst Biol. 2015 Feb 19;11(2):789. doi: 10.15252/msb.20145664 (PMC4358660; doi:10.15252/msb.20145664)
Supplement: Supplementary file 28 [file msb0011-0789-sd28.pdf]

molecular systems biology

Targeting a cell state common to Triple Negative Breast Cancers

Markus K Muellner, Barbara Mair, Yasir Ibrahim, Claudia Kerzendorfer, Hannelore Lechtermann, Claudia Trefzer, Freya Klepsch, André C Müller, Ernestine Leitner, Sabine Macho-Maschler, Giulio Superti-Furga , Keiryn L Bennett, Jose Baselga, Uwe Rix, Stefan Kubicek, Jacques Colinge, Violeta Serra and Sebastian MN Nijman

Corresponding author: Sebastian Nijman, CeMM Research Center for Molecular Medicine of the Austrian Academy of Sciences

Review timeline:

Submission date:	06 August 2014
Editorial Decision:	10 September 2014
Additional Correspondence	13 October 2014
Revision received:	09 December 2014
Editorial Decision:	07 January 2015
Revision received:	16 January 2015
Accepted:	22 January 2015

Editor: Maria Polychronidou

Transaction Report:

(Note: With the exception of the correction of typographical or spelling errors that could be a source of ambiguity, letters and reports are not edited. The original formatting of letters and referee reports may not be reflected in this compilation.)

1st Editorial Decision 10 September 2014

Thank you again for submitting your work to Molecular Systems Biology. We have now heard back from two of the three referees whom we asked to evaluate your manuscript. Since their recommendations are rather similar, I prefer to make a decision now rather than further delaying the process. As you will see from the reports below, the reviewers acknowledge that the presented findings are potentially interesting. However, they list a number of concerns and make suggestions for modifications, which we would ask you to carefully address in a revision of the manuscript.

Without listing all the points listed below, some of the more substantial issues raised by reviewer #1 are the following:
- Further analyses demonstrating the direct inhibition of SYK by PKC412 need to be included.
- The clinical relevance of the presented findings should be demonstrated.
- Additional controls, replicates and quantitative analyses are required in order to better support the main conclusions.

On a more editorial level we would like to encourage you to provide the Source Data for the figures/panels that show essential quantitative information. Additional information regarding source data is available in the "Author Guidelines" section in our website http://msb.embopress.org/authorguide#a3.4.

When you resubmit your manuscript, please download our CHECKLIST

© European Molecular Biology Organization

(<http://msb.embopress.org/sites/default/files/additional-assets/EMBO%20Press%20Author%20Checklist%20-MSB.xlsx>) and include the completed form in your submission. For convenience reasons, the CHECKLIST is also attached below.

If you feel you can satisfactorily deal with these points and those listed by the referees, you may wish to submit a revised version of your manuscript. Please attach a covering letter giving details of the way in which you have handled each of the points raised by the referees. A revised manuscript will be once again subject to review and you probably understand that we can give you no guarantee at this stage that the eventual outcome will be favorable.

REFEREE REPORTS

Reviewer #1:

Summary

In this article, Muellner et al. discovered a chemical inhibitor that displayed selective growth inhibition of triple negative breast cancer (TNBC) cells in vitro. This inhibitor, PKC412, displayed potent inhibition, pro-apoptotic and anti-tumor effects on subsets of TNBC and normal mammary epithelial cells as well as a patient-derived xenograft that displayed an EMT phenotype. Using a chemical proteomic screen, the authors go on to show that PKC412 selectively inhibited a subset of kinases including Aurora kinase A, FER tyrosine kinase, and SYK tyrosine kinase explaining the proposed mechanism of action of this new drug. To narrow down the target list, the authors performed a diffusion process algorithm that computationally predicts the probability of each PKC412 target, when removed, to perturb the enriched biological processes. Removal of SYK tyrosine kinase had a significant effect on the biological processes justifying further functional analysis of this kinase. The authors go on to provide functional data that genetic and pharmacologic SYK inhibition reduces TNBC viability but not luminal-like breast cancer cells in part through reduction in STAT3 activation.

Overall, the manuscript is well written, easy to follow, and provides intriguing data that co-targeting of SYK and Aurora Kinase A in TNBC may be a viable therapeutic opportunity clinically. The paper is stronger in terms of its chemical genetics than its systems biology. The data presented as is displays only indirect data on whether the primary target of PKC412 is indeed SYK, and direct inhibition of SYK is not measured. Without data supporting that SYK activity is directly diminished by PKC412 or that SYK and Aurora Kinase A are co-expressed in clinical tissues, the enthusiasm is somewhat diminished. Several critical data components are underpopulated, with problems such as no replicates, only one cell line of each class tested, no quantitation, lack of exact EC50 values being stated (Fig 2a). Without strong quantitative and statistical analysis, and the inclusion of additional lines and xenografts, several of the conclusions of the paper are currently tenuous. The manuscript could be made stronger by addressing the following points:

Major points that need to be addressed:

1. The critical Figure 4a is non quantitative, has only one replicate tested, has only one cell line tested in each category, and is thus inconclusive. Other figures also share this overall concern (Figures 5D, 1D), and others also suffer from only testing one cell from each category (Figure 6C).

2. To connect the authors' findings to the clinic, it would be helpful to show that SYK and Aurora kinase A protein and STAT3 phospho activation are co-occurring in TNBC and not in other breast cancer subtypes. The authors could do this via IHC or western blot analysis of clinical tissues. This would help to solidify their conclusions that this combination would be beneficial only in that subtype supporting the important concept of personalized medicine.

3. No negative control (luminal) tumor is used in the xenograft experiments. What if the drug is affecting, for example, tumor vasculature rather than the tumor?

4. Tumor growth was significantly affected based on the statistical analysis, but it is an overstatement to say it is a pronounced effect. It can be stated that the drug is slowing tumor growth,

but the relevance of this arguably minor effect in a mouse model in the context of cancer patient treatment is unclear.

5. The finding of PKC412 sensitivity of TNBC and the proposed kinase targets is informative. However, as the paper is written it is important to show that PKC412 is actually inhibiting SYK activity in cells. The authors present data in Supplemental Figure 11 that a different SYK inhibitor, R406, does indeed inhibit SYK activity in cells but do not show results for PKC412. While the authors have genetic data supporting SYK function in their models, and in vitro kinase activity assays for SYK inhibition by PKC412 (Fig. 4d), it would be important to also show that SYK phosphorylation is indeed reduced with this the new drug.

Minor points that need to be addressed:

1. Please include the phosphoresidues evaluated via western blot for both Aurora Kinase A and SYK in Supplemental Figures 10 and 11.

2. Can the authors comment on why a subset of TNBC cell lines are completely refractory to PKC412? This may be an interesting subtype within TNBC that PKC412 distinguishes or that has not been previously appreciated before or characterized.

3. The synergy score deviation from Bliss is not defined, explained in terms of how to interpret, nor is a reference provided.

4. The term non-oncogene addiction is used without any definition.

5. IN the discussion of the inhibitors, clarify if midostaurin is still considered broad spectrum in comparison to staurosporin.

6. Fig. 2c, only one sensitive line is tested in the apoptosis assay.

7. Specific numerical values should be quoted in the sentence "The EC50 for PKC412 that we observed for several cell lines was well below the reported plasma concentration in phase I trial patients22,23, indicating that this drug is active at a clinically achievable concentration."

8. No numerical values are provided in Fig. 2a. In the statement "Sensitivity varied dramatically across the cell line panel (Fig. 2a): several cell lines were refractory to PKC412 even at the highest concentration tested (50uM), whereas others were strongly inhibited and displayed half maximal effective concentration (EC50) values in the nanomolar range" the use of nanomolar range is too vague since this potentially covers three orders of magnitude. Similar issue for Supplementary Figure 1.

9. Fix the discrepancy between "whereas IKBKE (also known as IKK-epsilon) removal reduced the relationship only with apoptosis" and the figure that shows a change in the GO term "cell death" since cell death is a more general term than apoptosis.

10. "Our approach demonstrates that exploiting the transcriptomic landscape rather than the mutational landscape". The authors have not demonstrated that it is the transcriptomic landscape as opposed to some other regulatory level that is the main level of regulation responsible for the results they have observed.

11. Supplemental Fig. 11 legend says the dose used was 10 uM, but the figure annotation (triangle) indicates two different doses were used in some cases.

Reviewer #2:

The manuscript by Mueller et al. analyzes an impressive set of well presented experimental data to predict and validate some novel conclusions with regards to triple negative breast cancer (TNBC) therapy. Therapeutic options for TNBC are currently limited and it is one of the more deadly breast tumor types. They perform multiple large scale screening type experiments coupled with network

© European Molecular Biology Organization

modeling and detailed validation experiments to come to the quite surprising (and convincing) conclusion that SYK-involved with immune system regulation-is a viable drug target for some TNBCs. Moreover, there is not much experimental evidence that non-oncogene addictions are relevant for cancer therapy-this study helps to codify that general line of systems-based thinking that transcriptional state, rather than mutational state, based thinking can be useful for the field of cancer therapy. My only comments are quite minor, that the authors should make available all the data that went into this study in supplementary materials, and also that the authors might expand on the logic involved with the first screen, i.e. might the authors expect to have missed some potentially important drugs by requiring that the compounds were not toxic to MCF10A. That is, if a compound is effective against both 10A and 10A-twist cells, that doesn't necessarily mean it would not be promising for follow up with TBNC therapeutic options.

Additional Correspondence 13 October 2014

We have now received a late report from the third referee who was asked to evaluate your study.

The comments of this referee are included below for you reference. We would like to ask you to address this referee's concerns in the revision of your work and to include the related information in the point-by-point response.

Thank you once again for submitting your work to Molecular Systems Biology and we are looking forward to receiving your revised work.

REFEREE REPORT

Reviewer #3:

This manuscript identified a chemical compound, PKC412, that is capable of inducing apoptosis specifically in basal-like triple negative breast cancer cells by inhibiting AURKA and SYK. The authors began with a chemical screen in MCF-10A and Twist-overexpressing MCF-10A cells and found that breast epithelial cells that have undergone EMT are more sensitive to PKC412. Then, they found that basal-like breast cancer cells are more sensitive to PKC412. Furthermore, mechanistic studies suggest that SYK is a target of PKC412 in triple negative breast cancer cells and that AURKA and SYK inhibition synergistically kills basal-like breast cancer cells. This is an interesting study. However, several major weaknesses need to be addressed.

1. The authors started with a chemical screen and identified PKC412 as a compound that preferentially kills mesenchymal-like cells (MCF-10A with Twist overexpression) over epithelial cells (MCF-10A). However, in subsequent experiments, instead of showing the epithelial vs. mesenchymal state dictates the sensitivity of TNBC cells to PKC412, the authors focused on the correlation between the PCK412 sensitivity and the luminal vs. basal state. This is confusing.

2. According to Fig. 2A, not all basal-like TNBC cell lines are sensitive to PKC412; conversely, some of the luminal-like cell lines are sensitive to PKC412. Besides the luminal vs. basal state, are there any predictive markers that can be used to predict the sensitivity of PKC412? How to select patients that are likely to benefit from PKC412 treatment?

3. It is interesting that inhibition of SYK is able to kill certain basal-like breast cancer cells. However, SYK has been reported as a tumor suppressor or metastasis suppressor before, such as: Med Oncol. 2012 Jun;29(2):448-53 (Reactivation of Syk gene by AZA suppresses metastasis but not proliferation of breast cancer cells); PLoS One. 2009 Oct 15;4(10):e7445 (Tumor suppressor function of Syk in human MCF10A in vitro and normal mouse mammary epithelium in vivo); Nature. 2000 Aug 17;406(6797):742-7 (The Syk tyrosine kinase suppresses malignant growth of human breast cancer cells). How to address this discrepancy? This is important for deciding whether SYK should be targeted or not.

4. The authors claim that STAT3 is a downstream effector of SYK, but whether STAT3 mediates the function of SYK is not addressed. For example, can constitutively active STAT3 rescue the

© European Molecular Biology Organization

growth inhibition of breast cancer cells by PKC412 or SYK shRNA?
5. shRNA knockdown efficiency should be presented by western blots.

1st Revision - authors' response 09 December 2014

Response to reviewers (original comments are in ***italic/bold***)

Reviewer #1:

Summary
In this article, Muellner et al. discovered a chemical inhibitor that displayed selective growth inhibition of triple negative breast cancer (TNBC) cells in vitro. This inhibitor, PKC412, displayed potent inhibition, pro-apoptotic and anti-tumor effects on subsets of TNBC and normal mammary epithelial cells as well as a patient-derived xenograft that displayed an EMT phenotype. Using a chemical proteomic screen, the authors go on to show that PKC412 selectively inhibited a subset of kinases including Aurora kinase A, FER tyrosine kinase, and SYK tyrosine kinase explaining the proposed mechanism of action of this new drug. To narrow down the target list, the authors performed a diffusion process algorithm that computationally predicts the probability of each PKC412 target, when removed, to perturb the enriched biological processes. Removal of SYK tyrosine kinase had a significant effect on the biological processes justifying further functional analysis of this kinase. The authors go on to provide functional data that genetic and pharmacologic SYK inhibition reduces TNBC viability but not luminal-like breast cancer cells in part through reduction in STAT3 activation.

Overall, the manuscript is well written, easy to follow, and provides intriguing data that co-targeting of SYK and Aurora Kinase A in TNBC may be a viable therapeutic opportunity clinically. The paper is stronger in terms of its chemical genetics than its systems biology. The data presented as is displays only indirect data on whether the primary target of PKC412 is indeed SYK, and direct inhibition of SYK is not measured. Without data supporting that SYK activity is directly diminished by PKC412 or that SYK and Aurora Kinase A are co-expressed in clinical tissues, the enthusiasm is somewhat diminished. Several critical data components are underpopulated, with problems such as no replicates, only one cell line of each class tested, no quantitation, lack of exact EC50 values being stated (Fig 2a). Without strong quantitative and statistical analysis, and the inclusion of additional lines and xenografts, several of the conclusions of the paper are currently tenuous. The manuscript could be made stronger by addressing the following points:

In this revised manuscript we have addressed these matters and include new data showing *in vitro* and *in vivo* SYK inhibition by PKC412 and co-expression of SYK and AURKA in breast tumors. Furthermore, we have extended the experiments to additional cell lines and provide more quantification and statistical analysis. Together, we believe we have adequately addressed the raised concerns.

The data represented in Figure 2a (and Supplementary Figure 4) concerns rather large experiments with 28 cell lines (in several different culture media) 10 dose-points and duplicate measurements for each dose point. The value of this experiment is to compare relative sensitivities between the groups of cell lines. We feel that claiming absolute and definitive EC50 values for each individual cell line would be over interpreting the precision that can be obtained in such a large-scale approach. Despite this we have included EC50 calculations for PKC412 in Supplementary Figure 4. We have also performed more precise dose-response experiments on a smaller, representative panel of cell lines (Supplementary Figure 3).

Major points that need to be addressed:
1. The critical Figure 4a is non quantitative, has only one replicate tested, has only one cell line tested in each category, and is thus inconclusive. Other figures also share this overall concern (Figures 5D, 1D), and others also suffer from only testing one cell from each category (Figure 6C).

In addition to Figure 4a, we now provide new data with SYK knockdown in two additional luminal and basal cell lines and include a quantification and statistical analysis of this experiment (Supplementary Figure 12). Importantly, these new results are consistent with the notion that SYK inhibition is specifically cytotoxic to basal-like breast cancer cell lines.

We also provide quantification and statistical analysis of the experiments in Figures 5d (see new Supplementary Figure 14 and 15) and 1d (see new Supplementary Figure 1) and have extended the analysis in Figure 6c to one additional luminal and two basal-like cell lines.

2. To connect the authors' findings to the clinic, it would be helpful to show that SYK and Aurora kinase A protein and STAT3 phospho activation are co-occurring in TNBC and not in other breast cancer subtypes. The authors could do this via IHC or western blot analysis of clinical tissues. This would help to solidify their conclusions that this combination would be beneficial only in that subtype supporting the important concept of personalized medicine.

The reviewer correctly notes that SYK and AURKA need to be co-expressed in basal-like tumors for synergy to occur in this tumor type. We now provide evidence to show that both genes are ubiquitously expressed in TNBC (as a proxy for basal-like) breast tumors) (Supplementary Figure 20). As p-STAT3 has been previously demonstrated in basal like tumors (Marotta et al., JCI 2011; Buettner et al., Clin Can Res, 2002; Armanious et al., Int J Clin Exp Pathol 2010), it follows that SYK, AURKA and pSTAT3 indeed co-occur. These references are now included in the Discussion (page 12). Experimental evidence in cell lines in the manuscript also supports this notion.

However, the reverse logic that SYK, AURKA and pSTAT3 should not co-occur in luminal tumors does not automatically follow from this. Co-expression is not the cause of synergism, it is a requirement. Therefore, expression of these three proteins in luminal tissue is not in conflict with the notion that SYK/AURKA inhibition is synergistic only in a specific (i.e. basal-like) cell state. Experiments to understand in more detail why the SYK-STAT3 axis is specifically required in basal-like cells, is the topic of further investigation in the lab.

3. No negative control (luminal) tumor is used in the xenograft experiments. What if the drug is affecting, for example, tumor vasculature rather than the tumor?

The *in vitro* data already shows that the sensitivity of luminal cell lines is also variable in terms of response to PKC412. Thus, we do not think that this control would significantly improve the conclusion. A response of a luminal tumor does not necessarily refute our hypothesis and interpretation of data obtained (available luminal PDX tumors grow significantly slower than basal ones) would be difficult. Therefore, we think that there is no strong ethical justification to perform this experiment.

4. Tumor growth was significantly affected based on the statistical analysis, but it is an overstatement to say it is a pronounced effect. It can be stated that the drug is slowing tumor growth, but the relevance of this arguably minor effect in a mouse model in the context of cancer patient treatment is unclear.

We agree that the response is partial and does not result in tumor shrinkage. We have now indicated this in the text on page 6: "PKC412 inhibited tumor growth ($P<0.01$) in the CAL51 xenograft [ ]."

5. The finding of PKC412 sensitivity of TNBC and the proposed kinase targets is informative. However, as the paper is written it is important to show that PKC412 is actually inhibiting SYK activity in cells. The authors present data in Supplemental Figure 11 that a different SYK inhibitor, R406, does indeed inhibit SYK activity in cells but do not show results for PKC412. While the authors have genetic data supporting SYK function in their models, and in vitro kinase activity assays for SYK inhibition by PKC412 (Fig. 4d), it would be important to also show that SYK phosphorylation is indeed reduced with this the new drug.

We now include western blot analysis of p-SYK in PKC412 treated cells (Supplementary Figure 10)

Minor points that need to be addressed:
1. Please include the phosphoresidues evaluated via western blot for both Aurora Kinase A and

SYK in Supplemental Figures 10 and 11.

These changes have now been made in the corresponding figure legends (now Supplementary Figure 17 and 19).

2. Can the authors comment on why a subset of TNBC cell lines are completely refractory to PKC412? This may be an interesting subtype within TNBC that PKC412 distinguishes or that has not been previously appreciated before or characterized.

This is an interesting suggestion. We have found a tentative association with TP53 mutations but larger number of cell lines may be needed to establish this definitively. Alternatively, this could be related to the signaling and mutations upstream of STAT3. This is a topic that is under further study in the lab and we feel that this is too preliminary to include in this manuscript.

3. The synergy score deviation from Bliss is not defined, explained in terms of how to interpret, nor is a reference provided.

We now provide a formula of the Bliss synergy score in the Material and Methods on page 20 and refer to this in the text.

4. The term non-oncogene addiction is used without any definition.

We have added an explanation and reference for this term in this revised manuscript on page 4.

5. IN the discussion of the inhibitors, clarify if midostaurin is still considered broad spectrum in comparison to staurosporin.

Without a direct experimental comparison between midostaurin (PKC412) and staurosporin it is difficult to comment on this. It is certainly true that midostaurin inhibits multiple kinases, as is also evident from our chemical proteomics analysis. To more specifically point this out we have changed the text on page 5 to: "Like staurosporin, PKC412 is a multi-target protein kinase inhibitor and has been shown to have low nanomolar activity against additional kinases [ ]."

6. Fig. 2c, only one sensitive line is tested in the apoptosis assay.

We have expanded this analysis to two additional cell lines (Supplementary Figure 2). These experiments support our findings that PKC412 can induce apoptosis in basal like cell lines.

7. Specific numerical values should be quoted in the sentence "The EC50 for PKC412 that we observed for several cell lines was well below the reported plasma concentration in phase I trial patients22,23, indicating that this drug is active at a clinically achievable concentration."

We have adapted this sentence on page 6 to indicate the plasma concentration determined in patients: "The EC50 for PKC412 that we observed for several cell lines was well below the reported steady-state plasma concentration in phase I trial patients (0.2-0.7 μM)"

8. No numerical values are provided in Fig. 2a. In the statement "Sensitivity varied dramatically across the cell line panel (Fig. 2a): several cell lines were refractory to PKC412 even at the highest concentration tested (50uM), whereas others were strongly inhibited and displayed half maximal effective concentration (EC50) values in the nanomolar range" the use of nanomolar range is too vague since this potentially covers three orders of magnitude. Similar issue for Supplementary Figure 1.

We have adapted the text to: "[S]everal cell lines were refractory to PKC412 even at the highest concentration tested (50μM), whereas others were strongly inhibited.

9. Fix the discrepancy between "whereas IKBKE (also known as IKK-epsilon) removal reduced the relationship only with apoptosis" and the figure that shows a change in the GO term "cell death" since cell death is a more general term than apoptosis.

We thank the reviewer for pointing out this discrepancy and have adapted the text on page 9: "[ ] whereas IKBKE (also known as IKK-epsilon) removal reduced the relationship only with cell death. This suggests that inhibition of these kinases impinges on processes related to proliferation and cell death."

10. "Our approach demonstrates that exploiting the transcriptomic landscape rather than the mutational landscape". The authors have not demonstrated that it is the transcriptomic landscape as opposed to some other regulatory level that is the main level of regulation responsible for the results they have observed.

We have changed the wording to: "Our approach demonstrates that exploiting cell state rather than the mutational landscape of a subset of tumors can lead to the identification of novel treatment strategies."

11. Supplemental Fig. 11 legend says the dose used was 10 uM, but the figure annotation (triangle) indicates two different doses were used in some cases.

We thank the reviewer for pointing out this discrepancy and have adapted the text.

Reviewer #2:

The manuscript by Mueller et al. analyzes an impressive set of well presented experimental data to predict and validate some novel conclusions with regards to triple negative breast cancer (TNBC) therapy. Therapeutic options for TNBC are currently limited and it is one of the more deadly breast tumor types. They perform multiple large scale screening type experiments coupled with network modeling and detailed validation experiments to come to the quite surprising (and convincing) conclusion that SYK-involved with immune system regulation-is a viable drug target for some TNBCs. Moreover, there is not much experimental evidence that non-oncogene addictions are relevant for cancer therapy-this study helps to codify that general line of systems-based thinking that transcriptional state, rather than mutational state, based thinking can be useful for the field of cancer therapy.

My only comments are quite minor, that the authors should make available all the data that went into this study in supplementary materials,

In addition to the supplementary tables with the screening and proteomics data, we make the transcriptomics data available via Gene Expression Omnibus (GEO) under submission number GSE63721.

[ ] and also that the authors might expand on the logic involved with the first screen, i.e. might the authors expect to have missed some potentially important drugs by requiring that the compounds were not toxic to MCF10A. That is, if a compound is effective against both 10A and 10A-twist cells, that doesn't necessarily mean it would not be promising for follow up with TBNC therapeutic options.

We agree that compounds that kill both cell lines could be interesting to follow up. However, such compounds would also be enriched for generally toxic agents that would also kill normal cells. Therefore, we chose to not focus on these compounds. We now include a sentence in the text on page 4 to indicate this more clearly: "As compounds that killed both cell lines are enriched for general and unspecific toxic agents, we focused on compounds that were specifically toxic to MCF10A-Twist1 cells."

Reviewer #3:

This manuscript identified a chemical compound, PKC412, that is capable of inducing apoptosis specifically in basal-like triple negative breast cancer cells by inhibiting AURKA and SYK. The authors began with a chemical screen in MCF-10A and Twist-overexpressing MCF-10A cells and

found that breast epithelial cells that have undergone EMT are more sensitive to PKC412. Then, they found that basal-like breast cancer cells are more sensitive to PKC412. Furthermore, mechanistic studies suggest that SYK is a target of PKC412 in triple negative breast cancer cells and that AURKA and SYK inhibition synergistically kills basal-like breast cancer cells. This is an interesting study. However, several major weaknesses need to be addressed.

1. The authors started with a chemical screen and identified PKC412 as a compound that preferentially kills mesenchymal-like cells (MCF-10A with Twist overexpression) over epithelial cells (MCF-10A). However, in subsequent experiments, instead of showing the epithelial vs. mesenchymal state dictates the sensitivity of TNBC cells to PKC412, the authors focused on the correlation between the PCK412 sensitivity and the luminal vs. basal state. This is confusing.

Basal-like and claudin low breast tumors cells are enriched for mesenchymal features. However, for each single cell line a clear cut classification is lacking. Therefore, we chose to focus on cell lines of the basal-like type (including the claudin-low subtype) as a concensus classifcation for this is available. We modified the text on page 6 to point out the rationale more clearly.

2. According to Fig. 2A, not all basal-like TNBC cell lines are sensitive to PKC412; conversely, some of the luminal-like cell lines are sensitive to PKC412. Besides the luminal vs. basal state, are there any predictive markers that can be used to predict the sensitivity of PKC412? How to select patients that are likely to benefit from PKC412 treatment?

This is an interesting suggestion. We have found a borderline significance association with TP53 mutations and deletions but larger number of cell lines would be needed to establish this definitively. Alternatively, this could be related to the signaling and mutations upstream of STAT3. This is a topic that is under further study in the lab.

3. It is interesting that inhibition of SYK is able to kill certain basal-like breast cancer cells. However, SYK has been reported as a tumor suppressor or metastasis suppressor before, such as: Med Oncol. 2012 Jun;29(2):448-53 (Reactivation of Syk gene by AZA suppresses metastasis but not proliferation of breast cancer cells); PLoS One. 2009 Oct 15;4(10):e7445 (Tumor suppressor function of Syk in human MCF10A in vitro and normal mouse mammary epithelium in vivo); Nature. 2000 Aug 17;406(6797):742-7 (The Syk tyrosine kinase suppresses malignant growth of human breast cancer cells). How to address this discrepancy? This is important for deciding whether SYK should be targeted or not.

We are aware of the literature suggesting a tumor suppressor role of SYK in breast cancer. However, most of the data supporting a tumor suppressive role in breast cancer is from a single lab and SYK mutations have not been found in breast tumors. In our hands, chemical and genetic SYK inhibition resulted in decreased viability in various basal-like breast cancer cell lines. In addition to mentioning the proposed tumor suppressor role in the Introduction on page 4, we now again cite and mention the studies indicating a tumor suppressor role of SYK in breast cancer in the discussion on page 13:

"Interestingly, SYK has been implicated in the regulation of cell adhesion and migration, processes related to EMT, and suggested to act as a tumor suppressor in breast cancer. Together, this suggests that the role of SYK in supporting or suppressing mammary tumors may be highly context dependent and requires further investigation."

4. The authors claim that STAT3 is a downstream effector of SYK, but whether STAT3 mediates the function of SYK is not addressed. For example, can constitutively active STAT3 rescue the growth inhibition of breast cancer cells by PKC412 or SYK shRNA?

We thank the reviewer for this suggestion. However, we found that in our hands overexpression of constitutively active STAT3 resulted in toxicity making it difficult to measure effects of SYK inhibition.

5. shRNA knockdown efficiency should be presented by western blots.

We now include this in Supplementary Figure 11 (SYK) and 17 (STAT3).

© European Molecular Biology Organization

2nd Editorial Decision 07 January 2015

Thank you again for submitting your work to Molecular Systems Biology. We have now heard back from the referee who was asked to evaluate your manuscript. As you will see below, the referee is now satisfied with the modifications made. However, s/he lists a series of minor points that we would like to ask you to address in a revised version of the manuscript.

On a more editorial level we would like to ask you to deposit the phosphoproteomics data in one of the major public databases and to provide the dataset identifier in the "Data Availability" section of your manuscript. For more information you can refer to our journal policies on data deposition (http://msb.embopress.org/authorguide#a3.5>).

As a matter of course, please make sure that you have correctly followed the instructions for authors as given on the submission website.

Thank you for submitting this paper to Molecular Systems Biology.

REFEREE REPORTS

Reviewer #1:

The authors have provided adequate responses to the points raised with a few necessary minor revisions required.

1) The authors provided references to support pSTAT3 expression in these tumors and show gene expression data to support SYK and AURKA overexpression in TNBC tumors. The authors should plot Fig. S20 as a scatter plot of SYK versus AURKA expression levels to truly demonstrate cases of co-expression. To convey similar information as the current figure, TBNC data points can have a different color than non-TBNC data points.

2) For Figure 2a and Figure S3, provide the raw data so that others can estimate summary values (such as EC50 values) if these estimates are of use to them.

3) The authors add 2 additional cell lines to confirm their shRNA knockdown findings in Figure 4a, but need to indicate which shRNA was used in Fig. S12 for clarification.

4) Provide a reference for the Bliss synergy score.

5) Consider defining the addiction in a positive way rather than referring to it as a non-oncogene addiction.

2nd Revision - authors' response 16 January 2015

Point by point reply

- We would like to ask you to deposit the phosphoproteomics data in one of the major public databases and to provide the dataset identifier in the "Data Availability" section of your manuscript. For more information you can refer to our journal policies on data deposition (http://msb.embopress.org/authorguide#a3.5>).

We submitted the MS data to PRIDE and added the sentence "The mass spectrometry proteomics data have been deposited to the ProteomeXchange Consortium (http://proteomecentral.proteomexchange.org) via the PRIDE partner repository with the dataset identifier PXD001680." to the corresponding materials and methods section (p11).

© European Molecular Biology Organization

Reviewer #1:

The authors have provided adequate responses to the points raised with a few necessary minor revisions required.

1) The authors provided references to support pSTAT3 expression in these tumors and show gene expression data to support SYK and AURKA overexpression in TNBC tumors. The authors should plot Fig. S20 as a scatter plot of SYK versus AURKA expression levels to truly demonstrate cases of co-expression. To convey similar information as the current figure, TBNC data points can have a different color than non-TBNC data points.

This figure was now added to Supplementary Figure S20 and additional information was added to the Supplementary Figure Legends file.

2) For Figure 2a and Figure S3, provide the raw data so that others can estimate summary values (such as EC50 values) if these estimates are of use to them.

This data is now provided as Dataset 1 (Figure 2a) and Dataset 2 (Figure S3).

These datasets are now referenced in the text (p6 and p8).

3) The authors add 2 additional cell lines to confirm their shRNA knockdown findings in Figure 4a, but need to indicate which shRNA was used in Fig. S12 for clarification.

This information was now added to the Supplementary Legends File and stated more clearly in the Material and Methods section (p24).

4) Provide a reference for the Bliss synergy score.

The Reference [Bliss (1939), Ann. Appl. Biol. **26**:585-615."] has now been added to the references section of the manuscript.

5) Consider defining the addiction in a positive way rather than referring to it as a non-oncogene addiction.

We would prefer to maintain the term "Non-oncogene addiction" is an established concept in the field. See for example: Luo, J., Solimini, N. L., & Elledge, S. J. (2009). Principles of Cancer Therapy: Oncogene and Non-oncogene Addiction. *Cell*, *136*(5), 823–837. doi:10.1016/j.cell.2009.02.024
